# Basal Diet Fed to Recipient Mice Was the Driving Factor for Colitis and Colon Tumorigenesis, despite Fecal Microbiota Transfer from Mice with Severe or Mild Disease

**DOI:** 10.3390/nu15061338

**Published:** 2023-03-09

**Authors:** Daphne M. Rodriguez, Korry J. Hintze, Giovanni Rompato, Eliza C. Stewart, Abbey H. Barton, Emily Mortensen-Curtis, Porter A. Green, Arnaud J. Van Wettere, Aaron J. Thomas, Abby D. Benninghoff

**Affiliations:** 1 Department of Animal, Dairy and Veterinary Sciences, Utah State University, 4815 Old Main Hill, Logan, UT 84322, USA; 2 Department of Nutrition, Dietetics and Food Sciences, Utah State University, 8700 Old Main Hill, Logan, UT 84322, USA

**Keywords:** colitis, colorectal cancer, gut microbiome, fecal microbiota transfer, Western diet

## Abstract

Consumption of the total Western diet (TWD) in mice has been shown to increase gut inflammation, promote colon tumorigenesis, and alter fecal microbiome composition when compared to mice fed a healthy diet, i.e., AIN93G (AIN). However, it is unclear whether the gut microbiome contributes directly to colitis-associated CRC in this model. The objective of this study was to determine whether dynamic fecal microbiota transfer (FMT) from donor mice fed either the AIN basal diet or the TWD would alter colitis symptoms or colitis-associated CRC in recipient mice, which were fed either the AIN diet or the TWD, using a 2 × 2 factorial experiment design. Time-matched FMT from the donor mice fed the TWD did not significantly enhance symptoms of colitis, colon epithelial inflammation, mucosal injury, or colon tumor burden in the recipient mice fed the AIN diet. Conversely, FMT from the AIN-fed donors did not impart a protective effect on the recipient mice fed the TWD. Likewise, the composition of fecal microbiomes of the recipient mice was also affected to a much greater extent by the diet they consumed than by the source of FMT. In summary, FMT from the donor mice fed either basal diet with differing colitis or tumor outcomes did not shift colitis symptoms or colon tumorigenesis in the recipient mice, regardless of the basal diet they consumed. These observations suggest that the gut microbiome may not contribute directly to the development of disease in this animal model.

## 1. Introduction

The microbiome is a vast collection of microorganisms that live in association with the human body, including bacteria, viruses, fungi, and protists that reside on various surfaces, such as the skin, mouth, nose, lung, gastrointestinal tract, and genital tract [1]. The colon microbiome, which is composed of microbes that cohabit with each other and with local host cells, has an essential role in the host digestive system [2]. Gut homeostasis represents a state of equilibrium between the microbiome and host cells, resulting in a symbiotic or commensal association [3]. Microorganisms profit from a favorable environment and steady nutrient availability, and, in turn, these microorganisms support the host via microbial degradation of indigestible dietary components, stimulation of immune functions, production of certain vitamins, and other essential functions [4]. Understanding the dynamic relationship between the intestinal microbiome and gut health has prompted the development of murine and other animal models to further characterize microbiota profiles and their functionality.

Human and rodent gut microbiomes are largely similar and comprised of four dominant phyla: Firmicutes, Bacteroidetes, Actinobacteria, and Proteobacteria. The National Center for Biotechnology Information in the U.S. recently renamed these phyla as Bacillota, Bacteroidota, Actinomycetota, and Pseudomonadota, respectively, to improve systemic taxonomic nomenclature [5], although both designations are used synonymously nowadays. Modifiable lifestyle factors, such as the consumption of antibiotics, alcohol, or tobacco; the prevalence of chronic inflammatory disease; and diet can potentially change the gut microbiome [6]. The Western diet is characterized by high consumption of red meat, animal fat, and sugar, along with low fiber intake, and is considered a risk factor for many chronic diseases, such as inflammatory bowel disease (IBD) and colorectal cancer (CRC) [7]. The Western dietary pattern results in chronic deficiency of essential micronutrients, leading to a disruption of metabolic and biological pathways [8]. To investigate the interaction between the Western dietary pattern and the development of inflammation-associated colorectal cancer, we previously developed the total Western diet (TWD) for rodents based on the typical nutrient intake of Americans on an energy density basis [7]. In repeated studies, we had shown that chronic consumption of the TWD enhanced symptoms of colitis; increased inflammation and injury to colon mucosa; altered inflammation and immune signaling in mucosal tissues; and promoted colon tumorigenesis in a murine model of colitis-associated colorectal cancer (CAC) [9].

The involvement of the gut microbiome—considered either as a whole community of bacteria or in part with a focus on specific pathobionts—in the development of chronic intestinal inflammation and/or the development of colorectal cancer has been a subject of intense investigation in recent years. Importantly, individuals suffering from chronic colitis are at a two- to three-fold greater risk of developing CAC compared to healthy population [10]. IBD patients typically have a lower microbial load, reduced diversity in their gut microbiome, an elevated abundance of pro-inflammatory taxa, and greater quantities of bacteria belonging to the Erysipelotrichaceae and Streptococcaceae families (both within the Firmicutes phylum) [11]. Certain bacterial species have been shown to have pro-inflammatory properties by invading epithelial cells and inducing cytokine production, creating an ideal microenvironment for tumor development. Yu et al. reported that *Fusobacterium nucleatum* was abundant in the colorectal cancer tissues of CRC patients and suggested that it modulates innate immune signaling and promotes resistance to chemotherapy [12]. Furthermore, *F. nucleatum* selectively binds to E-cadherin, thereby increasing membrane permeability and activating the β-catenin signaling pathway, which upregulates the expression of oncogenic and pro-inflammatory genes in humans and mice [13]. Similarly, enterotoxigenic *Bacteroides fragilis*, a Gram-negative bacterium, triggers pathways leading to cleavage of E-cadherin, activation of the Wnt/β-catenin pathway and subsequent induction of the production of interleukin-17A and tumor necrosis factor; all of these collectively contribute to a pro-inflammatory tumor microenvironment [14]. Bonnet et al. reported that *Escherichia coli*, a Gram-negative bacterium belonging to the Enterobacteriaceae family, was more abundant in the tumor tissues obtained from CRC patients when compared to normal adjacent tissues, as well as when compared to the normal mucosa of control patients; of note, *E. coli* colonization was more pronounced in patients with advanced disease [15]. Mucosa-associated adhesive-invasive *E. coli* strains invade the mucosa, triggering the production of cancer-driving reactive oxygen species [15]. *Enterococcus faecalis*, a Gram-positive bacterium belonging to the Enterococcaceae family, promotes colon inflammation by generating extracellular superoxide and hydrogen peroxide, which leads to elevated DNA damage, increased expression of tumor growth factor-beta (TGFβ), activation of the SMAD signaling pathways, and upregulation of cyclooxygenase-2 [16]. Finally, *Peptostreptococcus anaerobius* (Clostridiaceae family), a Gram-positive oral bacterium generally found in mucosal tissue, was detected in high abundance in the stool samples and colon mucosa obtained from CRC patients; *P. anaerobius* was also shown to promote colon tumorigenesis in mice, enhance proliferation of human colon normal and cancer cell lines in vitro, and alter oncogenic pathways including cholesterol biosynthesis and TLR signaling, among others [17].

The mounting evidence pointing to an association, and perhaps a causal role, of gut bacteria in the pathogenesis of chronic inflammation and/or colon tumorigenesis has led to the development of multiple pre-clinical murine models in which the composition of the gut microbiome is manipulated to determine subsequent effects on gut health parameters. First among these is the gnotobiotic mouse, for which the composition of the microbiome is known. Generally, gnotobiotic (GB) mice are developed from a germ-free animal, either conceived via in vitro fertilization of a germ-free dam or birthed via cesarian section and then maintained in a pathogen-free environment. Then, the desired microbe(s) can be introduced via various means (e.g., co-housing, shared bedding, and inoculation) to colonize the germ-free mouse and establish a gnotobiotic model [18]. While the use of GB murine models is critical to understanding key microbial interactions within the gut, these models do have limitations in that they may lack important commensal resident bacteria that are essential for establishing gut homeostasis. Furthermore, laboratory mice bred in germ-free conditions develop anatomical, physiological, and immunological abnormalities [19]. In addition, GB colonies are expensive and difficult to maintain, and few facilities are available to sustain a germ-free environment. These limitations have led to the development of alternative models, including the use of antibiotics and antifungal drugs to deplete the resident microbiome of recipient mice prior to the transfer of fecal material from a donor animal in a process called fecal microbiota transfer (FMT) [20]. Hintze et al. depleted the gut microbiome of commonly used C57BL/6J mice with broad-scope antibiotics using this approach, followed by four weekly transfers of fecal material from two human donors; the authors found that the recipient mice’s microbiomes reflected approximately 68 to 75% of the human microbiome sequence mass [20]. We employed a similar antibiotic approach in a human-to-mouse FMT experiment in which fecal material from either obese or lean human donors was transferred to mice fed either a healthy diet, a high-fat diet, or a complete Western diet [21]. Interestingly, we found that the diet fed to the recipient mice was the driving force in shaping the gut microbiome of the recipient mice as opposed to the source of the donated microbiota. The recipient mice that received FMT from obese human donors and were fed a standard diet did not acquire an overweight phenotype, nor did FMT from lean donors protect the mice that were fed a high-fat diet from gaining excess weight [21]. These findings point to the critical need to consider the diet fed to recipient mice when performing FMT experiments to determine if gut bacteria from donors may confer host traits to recipients.

While multiple studies have pointed to gut dysbiosis as a common feature of colitis and/or colon tumorigenesis in humans and animal models, it is not clear whether changes in the gut microbiome composition associated with different nutritional patterns are the drivers of this disease process, leading to the progression from initial inflammation to neoplasia and then advanced tumorigenesis. Therefore, to better understand the involvement of the microbiome in the development and/or exacerbation of colitis and colon tumorigenesis, we designed an FMT experiment using fecal material collected from a prior study, in which donor mice were fed either the AIN93G diet or the TWD and subjected to our standard protocol for mouse CAC [22]. This experimental design incorporated the standard AIN93G diet or the total Western diet for the donor animals as well as for the recipient mice in a 2 × 2 factorial design. We hypothesized that FMT from the donor mice that were fed the TWD and experienced severe colitis and high tumor burden would exacerbate symptoms of colitis and increase tumorigenesis in the recipient mice that were fed the AIN93G diet. Conversely, we hypothesized that FMT from the donor mice that were fed the AIN93G diet and had mild colitis and low tumor burden would alleviate colitis symptoms and reduce tumorigenesis in the recipient mice that were fed the TWD.

## 2. Materials and Methods

### 2.1. Chemicals and Reagents

Azoxymethane (AOM) was purchased from Sigma-Aldrich (St. Louis, MO, USA; CAS No. 25843-45-2). Dextran sodium sulfate (DSS; reagent grade at mol. Wt. ~40 kDa) was obtained from Thermo Fisher Scientific (Waltham, MA, USA). All other chemicals were obtained from general laboratory suppliers at reagent grade. Other reagents and kits are described below.

### 2.2. Experimental Animals

The Utah State University Institutional Animal Care and Use Committee approved all procedures for the handling and treatment of mice used for this study (protocol no. 11562). The husbandry procedures and facilities for housing the mice were exactly the same as described in a previous study [22].

### 2.3. Collection of Fecal Material from Donor Mice

The principal research question of this study is whether the fecal microbiome associated with a Western diet enhances colitis symptoms, colon tumorigenesis, and microbiome modulation, when it is transferred into recipient mice fed either a healthy diet (AIN93G) or the total Western diet (TWD). Fecal material was collected weekly in a previous study [22] from mice that were fed either the AIN basal diet or the TWD in a longitudinal study employing the AOM + DSS model of chemical carcinogenesis (Figure S1a) and was used for FMT to recipients, as outlined below. The fecal samples were collected and stored in a −80 °C freezer. To prepare the material for FMT, these fecal samples were pooled by basal diet and experimental week (Figure S1a) and diluted to 1 g/mL in sterile saline one week prior to their use for FMT, and stored at −20 °C. On the day of transfer, the samples were thawed on ice and then used for oral gavage, as described below.

### 2.4. Experimental Diets for Recipient Mice

The experimental diets were formulated by Envigo (Hackensack, NJ, USA; formerly Harlan Teklad) as outlined in Table S1; they were obtained from the vendor as one lot and maintained at 4 °C for the duration of the study. The two basal diets were the AIN93G diet (AIN, cat. No. TD.94045), formulated to promote rodent health with an energy density of 3.8 kcal/g, and the total Western diet (TWD, cat. No. TD.180497), the formulation of which was previously published [7], with an energy density of 4.4 kcal/g, and which was designed to emulate typical macro- and micronutrient intakes on an energy density basis. The diets were administered, and food intake was monitored as previously described [22].

### 2.5. Microbiota Depletion and Fecal Microbiota Transfer from Mouse Donors

The depletion of the recipient mice’s resident microbiome was achieved using a previously described protocol [20]. Twelve hours after the last antibiotic treatment, each mouse was dosed by oral gavage with the fecal matter diluted in sterile saline (1 g/mL) from its assigned FMT source group, which included either AIN-fed or TWD-fed donor mice. FMT continued weekly in a time-matched fashion (Figure S1b) until the end of the study.

### 2.6. Experimental Design

A 2 × 2 factorial design was employed with the basal diet fed to the FMT recipients (referred to henceforth as “basal diet”) and the basal diet fed to the FMT donors (henceforth, “FMT source”) as the two main experimental factors, resulting in the following experimental groups (basal diet/FMT source): (1) fed AIN/fmt AIN, (2) fed AIN/fmt TWD, (3) fed TWD/fmt AIN, and (4) fed TWD/fmt TWD (Figure S1b). The mice were assigned to one of these experimental groups at 5 weeks of age using a random block design to standardize body weights among the experimental groups. The recipient mice were provided one of the two basal diets, AIN93G (group 1–2) or TWD (group 3–4), for 3 days before starting the antibiotic regimen.

The protocol for inducing colitis and colon tumorigenesis had been described in a previous study [22] and was followed in this experiment with only slight modifications. On day 14, the mice were dosed *i.p.* with 10 mg/kg of AOM prepared in sterile PBS and provided 1% (*w/v*) DSS via drinking water for 10 days, followed by plain drinking water for the remainder of the experiment. On days 26 and 38, the mice were temporarily placed in new cages blinded to treatment, and the DAI score was determined as described in a previous study [9]. Additionally, on days 26 and 38, a randomly selected subset of mice from each group (*n* = 7 to 11 per group) was euthanized by CO_2_ asphyxiation and necropsied, as outlined in a previous study [9]. The histopathological assessment of epithelial inflammation and mucosal injury was performed by a board-certified veterinary pathologist at the Utah Veterinary Diagnostic Laboratory using a scoring system as previously described [9]. On day 105, body composition was determined for all mice using an MRI scan (EchoMRI-700). On day 115, the remaining mice (*n* = 22 to 27 per group) were euthanized by CO_2_ asphyxiation and necropsied, as described in a previous study [9]. A randomly selected subset of colon tissues (*n* = 13 to 17 per group) was preserved for histopathological verification of cancer stage.

### 2.7. Microbiota Profiling by 16S rRNA Sequencing

Sequencing of fecal microbiome was performed using the MiSeq reagent kit v2 for a paired-end 500 cycle (2 × 250 bp) (Illumina, San Diego, CA, USA), as previously described [22]. Fresh fecal samples were collected by cage on day 14 (pre-DSS), day 26 (colitis), day 38 (recovery), and day 115 (terminal), and stored at −80 °C until analysis. The methods for DNA isolation, amplification, purification, quantitation, and sequencing were as previously outlined [22]. The microbiota sequences were processed using QIIME 2 [23] and DADA2 [24]. Briefly, the DADA2 R package with a full amplicon workflow, including filtering, dereplication, chimera identification, and merging paired-end reads, generated an amplicon sequence variant (ASV) table and representative sequences. To assign taxonomy, the Qiime feature-classifier classify sklearn command was used with a pre-trained classifier for the v4 region, silva-138-99-515-806-nb-classifier.qza, and the most recent release of the SILVA database [25]. Supplementary File S1 provides the resulting count data collapsed to the family level.

### 2.8. Microbiome Sequencing Data Analysis

The sequence data were analyzed using the Microbiome Analyst Marker Data Profiling module [26] as previously described [22]. The data were analyzed for the main effects of *basal diet* and *FMT source*, as well as for selected a priori pair-wise comparisons as follows: (1) fed TWD/fmt AIN vs. fed AIN/fmt AIN (effect of basal diets in mice given fmt from AIN-fed donors); (2) fed TWD/fmt TWD vs. fed AIN/fmt TWD (effect of basal diets in mice given fmt from TWD-fed donors); (3) fed AIN/fmt TWD vs. fed AIN/fmt AIN (effect of fmt from different donors on mice fed the AIN basal diet); and (4) fed TWD/fmt TWD vs. fed TWD/fmt AIN (effect of fmt from different donors on mice fed the TWD). Alpha diversity (number ASVs, Chao1 richness, and Shannon index) and beta diversity (unweighted and weighted unifrac distances) were assessed as described in a previous study [22]. A permanova *p*-value < 0.01 for β-diversity was considered statistically significant. The taxonomic relative abundance data were analyzed using the metagenomeSeq with a zero-inflated Gaussian fit, and a false discovery rate-adjusted *p*-value < 0.05 was considered statistically significant. Clustvis was used to perform unsupervised, bidirectional hierarchical cluster analyses using the relative abundance data at the family taxonomical level [27]. Heat trees representing the hierarchical structure of the taxonomic classifications were generated for the pairwise comparisons listed above (for the non-parametric Wilcoxon rank-sum test, a *p*-value < 0.05 was considered significant).

### 2.9. Statistical Analysis

Statistical analyses for tumor incidence were performed using the Fisher’s exact test, followed by a Bonferroni adjustment to correct for multiple testing (Prism v. 8, GraphPad Software, San Diego, CA, USA). Other data were analyzed using a generalized linear mixed model (GLMM) with cage as a nested, random factor and using the restricted maximum likelihood (REML) estimation and Tukey’s HSD post hoc test for multiple comparisons (JMP v.16.2.0, SAS Institute, Cary, NC, USA). The main effects of basal diet and FMT source, and their interaction, were determined for each time point. Suspected outliers were verified using the robust outlier test (ROUT) with a conservative Q value of 1% (Prism), meaning that there is a ≤1% chance of excluding a data point as an outlier in error. Data that did not meet the equal variance assumption were log_10_ or square root transformed. For data that were not normally distributed or for which a transformation did not equalize variance, a nonparametric Steel–Dwass test was employed (JMP) to assess the main effects of diet and FMT source (no interaction test possible). However, if the results of the nonparametric Steel–Dwass tests were not different from the original GLMM analyses with respect to significant outcomes, the original GLMM test results were reported because the mixed model accounts for potential cage effects. A significant effect of the test variable was inferred when the adjusted *p*-value was <0.05.

## 3. Results

### 3.1. Food and Energy Intakes, Body Weight and Composition, and Organ Weights

**Figure 1:**
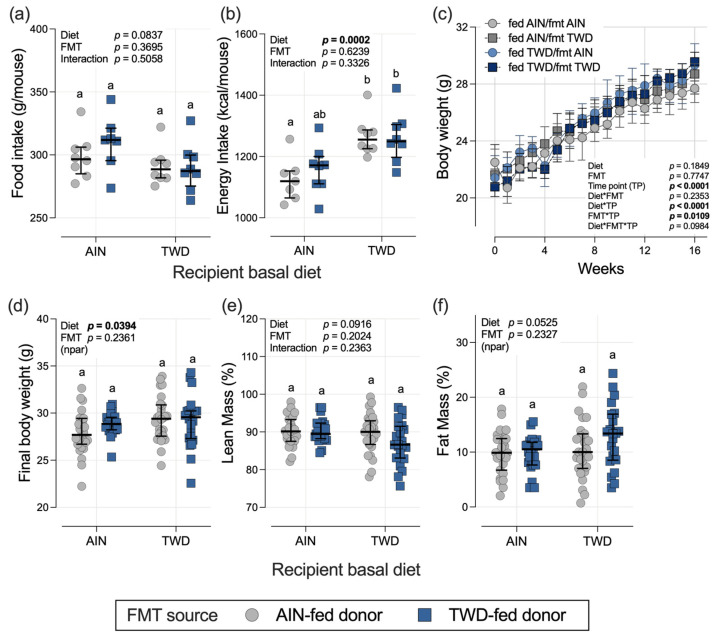
Food and energy intakes, body weight, and body composition. (**a**,**b**) Estimated total daily food and energy intakes per mouse per cage (*n* = 7 to 8 cages per group). (**c**) Body weight gain over the study period (*n* = 22 to 27 mice per group). (**d**) Final body weight at the study end point on day 112 (*n* = 22 to 27 mice per group). (**e**,**f**) Lean and fat mass as percentage of body weight. (*n* = 22 to 27 mice per group). The data are shown as individual measurements (except (**c**)) with the median ± interquartile range (**a**,**b**,**d**–**f**). The inserted values show the statistical model’s main effects for recipient basal diet and FMT source, and their interaction, or “npar” if a non-parametric test was required, and different letters indicate that the experimental groups are significantly different (*p* < 0.05), as determined by the statistical methods outlined in the Materials and Methods section.

Total food intake for the study period was not significantly different among the diet or FMT source groups, whereas energy intake was significantly higher (9.6% overall) in the recipient mice fed the TWD compared to those fed the AIN diet (diet main effect, *p* = 0.0002), reflecting the higher energy density of the TWD (Figure 1a,b). This increased energy intake led to a small but significant increase in final body weight of 3.3% in the recipient mice fed the TWD (diet main effect, *p* = 0.0394) (Figure 1c,d). However, the basal diet fed to the recipient mice did not significantly alter either lean or fat mass composition (Figure 1e,f), although liver, spleen, and kidney weights were all higher in the TWD-fed recipients (Figure S2). Of note, FMT from the TWD-fed donor mice, when compared to the AIN-fed donors, did not significantly affect food intake, energy intake, body weight gain, lean or fat mass composition, relative liver weight, relative kidney weight, or cecum content (Figure 1 and Figure S2). However, relative spleen weight in the TWD-fed recipients that received FMT from the TWD-fed donors was significantly reduced compared to their counterparts that received FMT from the AIN-fed donors (Figure S2b). The relative mass of cecum contents was not affected by diet or FMT donor source (Figure S2d).

### 3.2. Symptoms of Colitis and Histopathological Scoring

**Figure 2:**
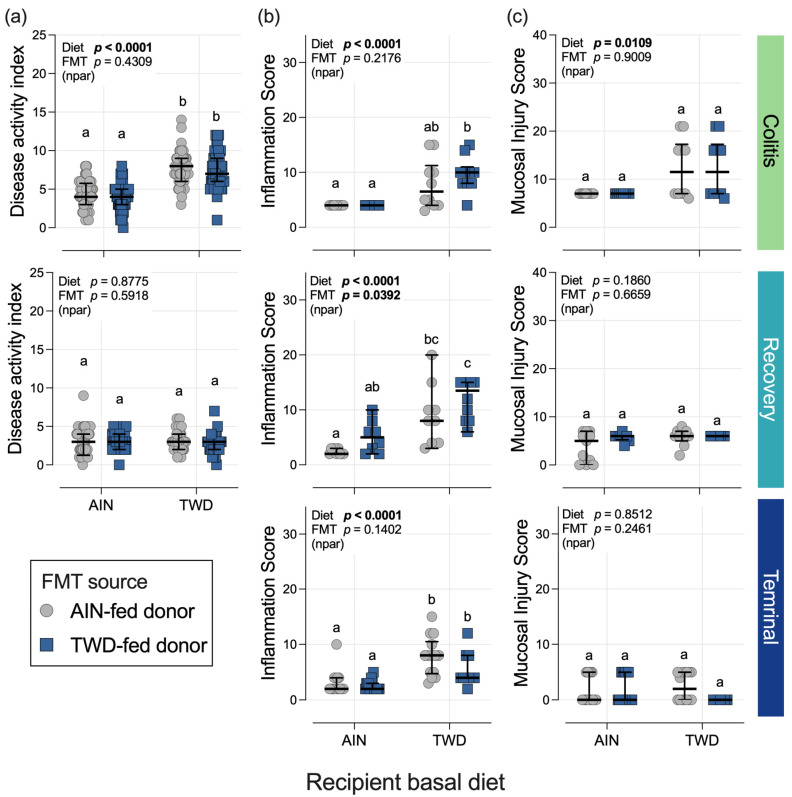
Disease activity index and colon histopathology. Scores for the disease activity index (DAI) (**a**), mucosal inflammation (**b**), and mucosal injury (**c**) are shown for active colitis on day 26, for recovery from gut injury on day 38, and for the terminal time point on day 112. The data are shown as individual values with median ± interquartile range. For the DAI score, *n* = 53 to 64 mice per group at the active colitis time point and *n* = 37 to 44 mice per group at the recovery time point. For inflammation and mucosal injury scores, *n* = 10 to 11 mice per group at the active colitis time point, *n* = 7 to 11 mice per group at the recovery time point, and *n* = 10 to 16 mice per group at the terminal time point. The inserted values provide the statistical model’s main effects for recipient basal diet and FMT source, and their interaction, or “npar” if a non-parametric test was required, and different letters indicate that the experimental groups are significantly different (*p* < 0.05), as determined by the statistical methods outlined in the Materials and Methods section.

Compared to the recipient mice fed the AIN diet, consumption of the TWD increased symptoms of colitis, as measured by the DAI score, by 1.8-fold during active colitis on day 26, although this elevation during active disease did not persist through the recovery phase in this study (Figure 2a). However, FMT from the AIN- or TWD-fed donor mice did not alter colitis symptoms at either time point. The histopathological assessment of colon inflammation and mucosal injury indicated a strong promoting effect of the TWD on the recipient mice during active colitis (diet main effect, *p* < 0.0001 and =0.0109, respectively), with the prolonged elevation of colon inflammation in the TWD-fed mice persisting through recovery (diet main effect, *p* < 0.0001) and to the terminal time point at day 112 (diet main effect, *p* < 0.0001) (Figure 2b,c). However, the mucosal injury had largely resolved by the recovery time point, with no further apparent effect of the TWD. For colitis symptoms and mucosal injury, FMT from the donor mice fed either the AIN diet or the TWD did not have any apparent significant effects (Figure 2). However, a significant main effect of FMT from the donor mice fed the TWD was noted for inflammation scores at the recovery time point (*p* = 0.0392), suggesting that the microbiota transferred from previously TWD-fed mice exacerbated colon inflammation well into recovery due to DSS-induced gut injury (Figure 2b).

### 3.3. Colon Length and Tumorigenesis

**Figure 3:**
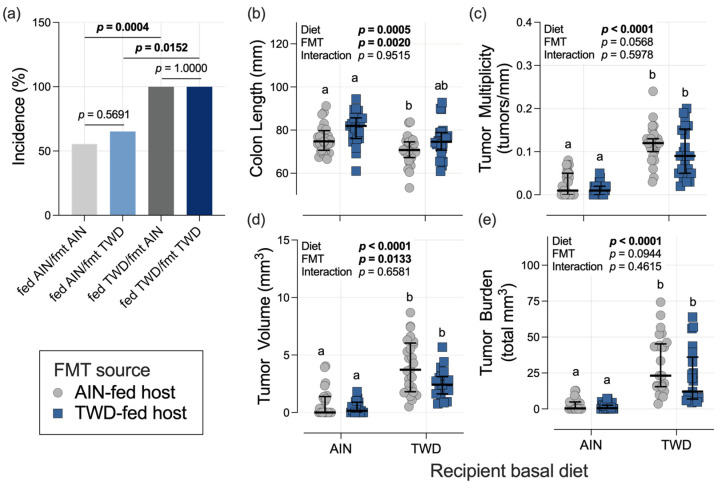
Effect of recipient basal diet and FMT source on colon length and colon tumorigenesis. (**a**) Incidence of colon tumors shown as the percentage of mice with tumors at the terminal time point (*n* = 22 to 27 mice per group). *p*-values from pairwise Fisher’s exact tests (selected a priori) are shown. (**b**) Colon length (*n* = 22 to 27 mice per group). (**c**) Colon tumor multiplicity (number of tumors per mm colon length) (*n* = 19 to 27 mice per group). (**d**) Average tumor volume (*n* = 18 to 25 mice per group). (**e**) Tumor burden (total volume) (*n* = 20 to 25 mice per group). For (**b**–**e**), the data are shown as individual values with median ± interquartile range. The inserted values provide the statistical model’s main effects for recipient basal diet and FMT source, and their interaction, and different letters indicate that the experimental groups are significantly different (*p* < 0.05), as determined by the statistical methods outlined in the Materials and Methods section.

The basal diet fed to the recipient mice significantly altered the incidence of colon tumors. Specifically, for mice that received FMT from the AIN-fed donors, tumor incidence in the TWD-fed recipients was 100% compared to only 56% for their AIN-fed counterparts (*p* = 0.0004); similarly, for mice that received FMT from the TWD-fed donors, tumor incidence was 100% versus 65% for the AIN-fed recipients (*p* = 0.0152) (Figure 3a). Alternatively, within each basal diet group, FMT from either the AIN- or TWD-fed donors did not alter tumor incidence.

In the mice that were necropsied at the study end point, the average colon length of the recipient mice was affected by both experimental factors, namely the basal diet fed to the recipient and the FMT donor source (Figure 3b). First, when considering only the effect of basal diet, colon length was 7.0% shorter in the TWD-fed recipients compared to their AIN-fed counterparts (diet main effect, *p* = 0.0005). Next, considering the effect of the FMT source, the colons of the mice that received FMT from the TWD-fed donors were 6.4% longer on average (FMT source main effect, *p* = 0.0020). However, no interaction between basal diet and FMT source was noted, indicating that the effect of FMT was consistent regardless of the basal diet fed to the recipient mice.

As expected, the recipient mice fed the TWD experienced a 5-fold increase in tumor multiplicity, a 5-fold increase in tumor volume, and an 11-fold increase in tumor burden (diet main effect, *p* < 0.0001), irrespective of FMT donor (Figure 3c–e). FMT from the AIN- or TWD-fed donors did not significantly alter tumor multiplicity or tumor burden, and FMT from the TWD-fed donors appeared to reduce average tumor volume (FMT source main effect, *p* = 0.0133); however, this effect was not dependent on the basal diet as there was no interaction between FMT source and basal diet (*p* = 0.6581) (Figure 3d).

### 3.4. Fecal Microbiome Response to FMT Source and Basal Diet

After fecal bacterial DNA isolation, a total of 12.3 × 10^6^ amplicons were sequenced and filtered for length, quality, and chimeras, leaving 8.3 × 10^6^ total sequences for an average of 38,713 sequence reads per sample assigned to 3101 ASVs. The sequencing depth was set to ~4444 sequences for diversity analyses (Figure S3).

**Figure 4:**
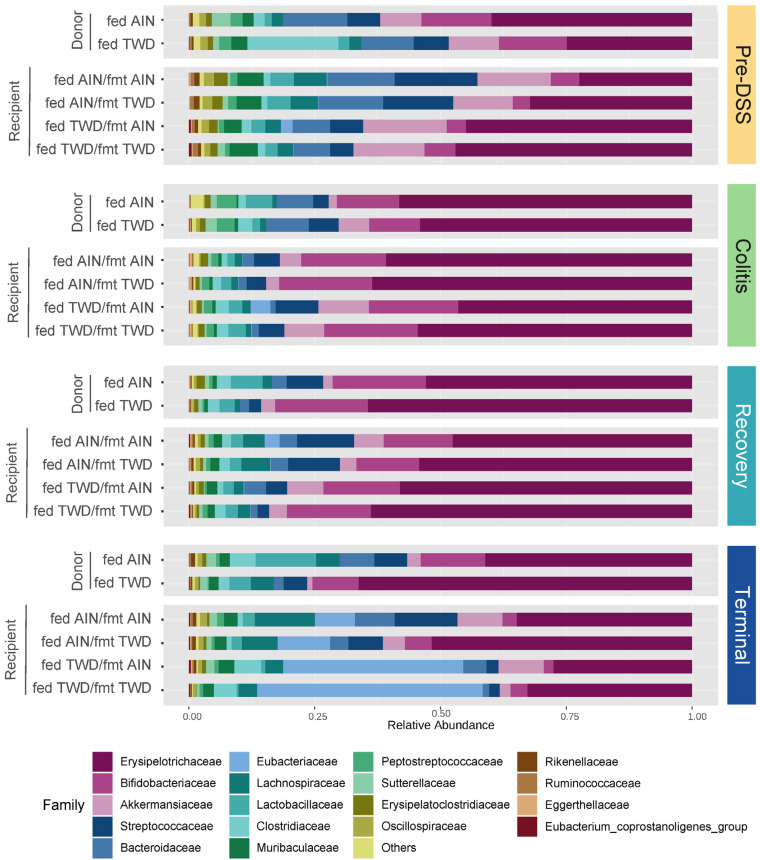
Taxonomic classification of mouse fecal bacteria. The data shown are the average relative normalized abundance of bacteria annotated to the family taxonomic level for the top 18 most abundant taxa for each experimental group at each time point (*n* = 19 to 20 cages per group at the pre-DSS time point, *n* = 17 to 18 cages per group at the active colitis time point, *n* = 12 to 13 cages per group at the recovery time point, and *n* = 7 to 8 cages per group at the terminal time point). The donor fecal microbiome sequence data are obtained from Rodriguez et al. [22]. However, the donor sequence data were reprocessed in conjunction with the recipient fecal microbiome sequence data in this study so that the normalization for both donor and recipient data sets was consistent. The data for the phylum taxonomic level are available in Figure S4.

**Figure 5:**
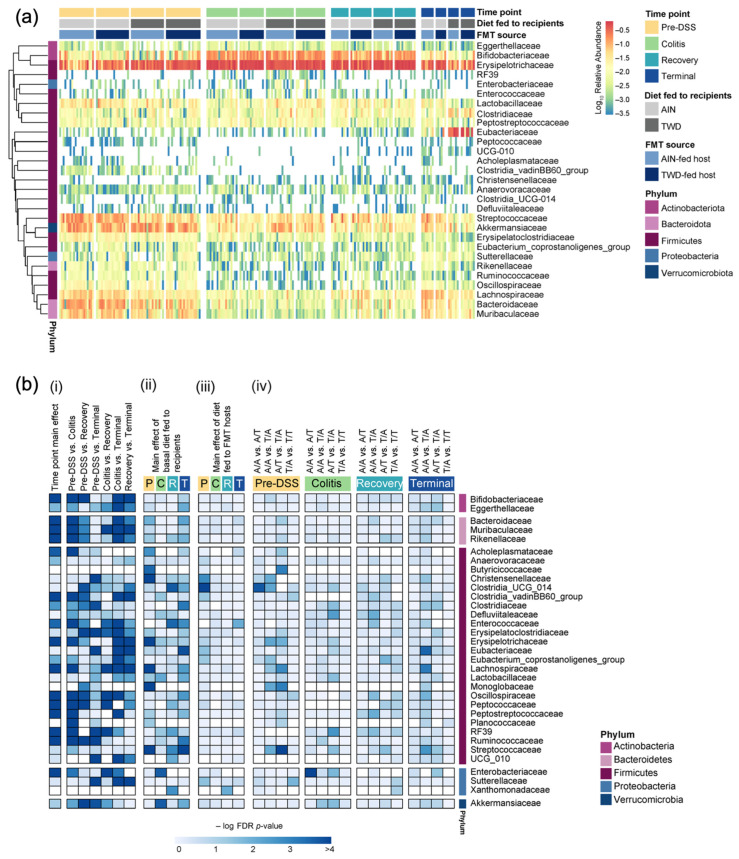
Relative abundance of fecal microbiome at the family taxonomic level with a summary of the results from the metagenomeSeq statistical analyses. (**a**) Unsupervised hierarchical cluster analysis shows the log_10_ relative abundance with clustering by taxa using the Euclidean distance with average linkage (*n* = 19 to 20 cages per group at the pre-DSS time point, *n* = 17 to 18 cages per group at the active colitis time point, *n* = 12 to 13 cages per group at the recovery time point, and *n* = 7 to 8 cages per group at the terminal time point). (**b**) Summary plot shows the log_10_ FDR-adjusted *p*-values obtained from the metagenomeSeq analyses of fecal microbiome profiles. All tests were determined a priori, and the complete results are provided in Supplementary File S2. (i) Analyses for the main effects of time point and pairwise comparisons across time points, irrespective of basal diet or FMT source. (ii) Analyses for diet main effects, irrespective of FMT source, at each time point. (iii) Analyses for FMT source main effects, irrespective of basal diet, at each time point. (iv) Selected pairwise tests for basal diet and FMT source combinations within each study time point. The abbreviations for the recipient basal diet/FMT source are as follows: A/A, fed AIN/fmt AIN; A/T, fed AIN/fmt TWD; T/A, fed TWD/fmt AIN; and T/T, fed TWD/fmt TWD. A significant effect is inferred for FDR-adjusted *p*-values < 0.05 (increasing blue on the color scale).

This experimental design included multiple factors to explore the dynamics of the mouse gut microbiome after antibiotic depletion of resident microbiota, followed by FMT from the donor mice and then the standard AOM + DSS regimen to induce colitis and colon tumorigenesis in the mice fed either the standard AIN diet or the TWD. Thus, the statistical analyses were performed in a stepwise manner, considering first the overall effect of the study time point, and then the effects of basal diet and FMT source within each time point. First, the taxonomic composition of the fecal microbiome was greatly affected by the chemical induction of colitis, as indicated by an overall increase in the relative abundances of Erysipelotrichaceae (primarily *Dubosiella newyorkensis* and *Turicibacter* spp.) from 41% to 57% (*p* = 0.0001) and Bifidobacteriaceae (*Bifidobacterium* spp.) from 3.4% to 16.9% (*p* < 0.0001) in the fecal microbiome, regardless of the basal diet or FMT source, whereas the relative abundances of other taxa decreased, such as Akkermansiaceae (*Akkermansia muciniphila*) from 13.4% to 5.6% (*p* = 0.0067), Streptococcaceae (*Lactococcus* spp.) from 11.8% to 6.6% (*p* = 0.0216), Lachnospiraceae (primarily *Lachnoclostridium*, *A2*, *Marvinbryantia,* and other unclassified genera) from 4.4% to 1.4% (*p* ≤ 0.0001), and Bacteroidaceae (*Bacteroides* spp.) from 9.6 to 1.2% (*p* < 0.0001) (Figure 4 and Figure 5, Supplementary Figures S4 and S5 and File S2). These shifts in relative abundance persisted for the most part through the recovery phase, although the abundance of Lachnospiraceae increased from 1.2% at the colitis time point to 4.5% at recovery (*p* < 0.0001), showing a level similar to the pre-DSS abundance of 3.8% (Figure S5). However, the fecal microbiome at the terminal time point was distinct with a notable marked increase in Eubacteriaceae, comprising 26% of the microbiome on average (*p* < 0.0001 for all time points), irrespective of the basal diet or FMT source. This increase in Eubacteriaceae corresponded to a proportional decrease of 20% for Erysipelotrichaceae (*p* < 0.0001) and of 10.5% for Bifidobacteriaceae (*p* < 0.0001) at the terminal time point compared to recovery, so that their relative abundance was more similar to that prior to DSS-induced gut injury. Additionally, of note, the relative abundance of Sutterellaceae (*Parasutterella uncultured_organism)* was different over the course of disease development, with the relative abundance of this taxon being significantly different at the pre-DSS (0.82% of the population), colitis (0.37%), and recovery (0.42%) time points when compared to the terminal time point (1.15%) (*p* < 0.0001 for all comparisons to the terminal time point) (Figure S5).

**Figure 6:**
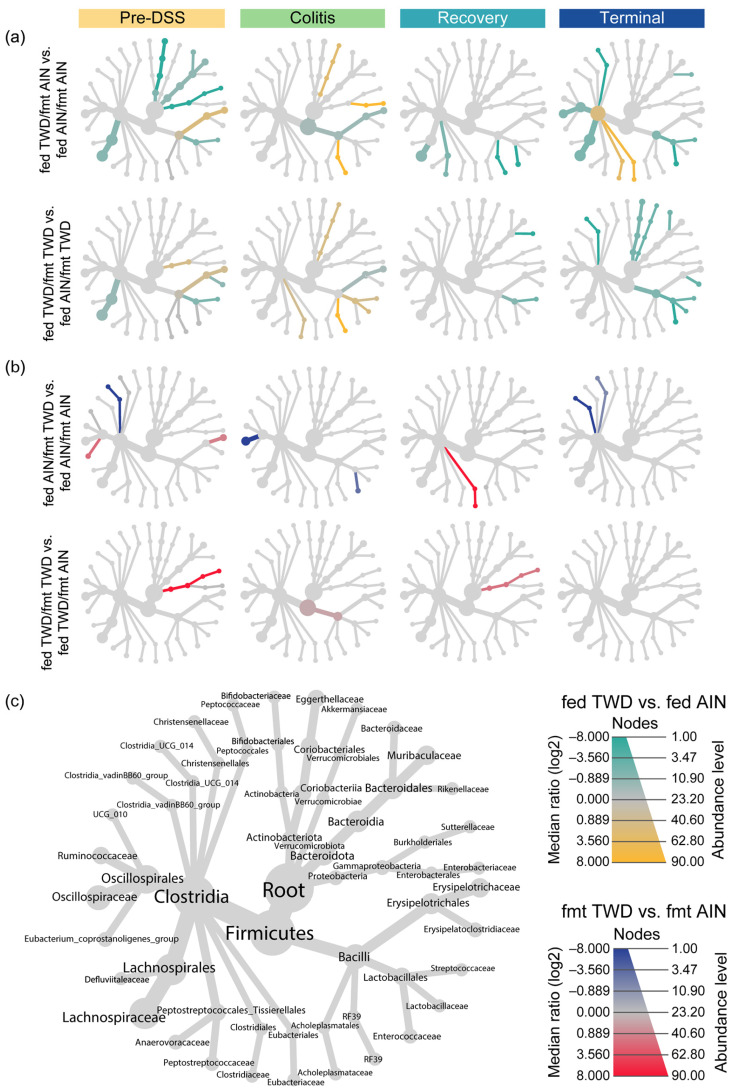
Fecal microbiome community structures depicted as heat trees, showing the relative abundance ratios for selected comparisons of recipient basal diet and FMT source at each experimental time point. (**a**) Comparisons of effects of recipient basal diet on mice receiving FMT from either an AIN-fed or a TWD-fed donor (green-to-yellow color bar, with yellow indicating greater abundance in the TWD-fed mice; top legend). (**b**) Comparisons for effects of FMT source on recipient mice fed either the AIN basal diet or the TWD (blue-to-red color bar, with red indicating greater abundance in FMT from the TWD-fed donors; bottom legend). (**c**) Phylogenetic structure of fecal microbiome bacterial community.

**Figure 7:**
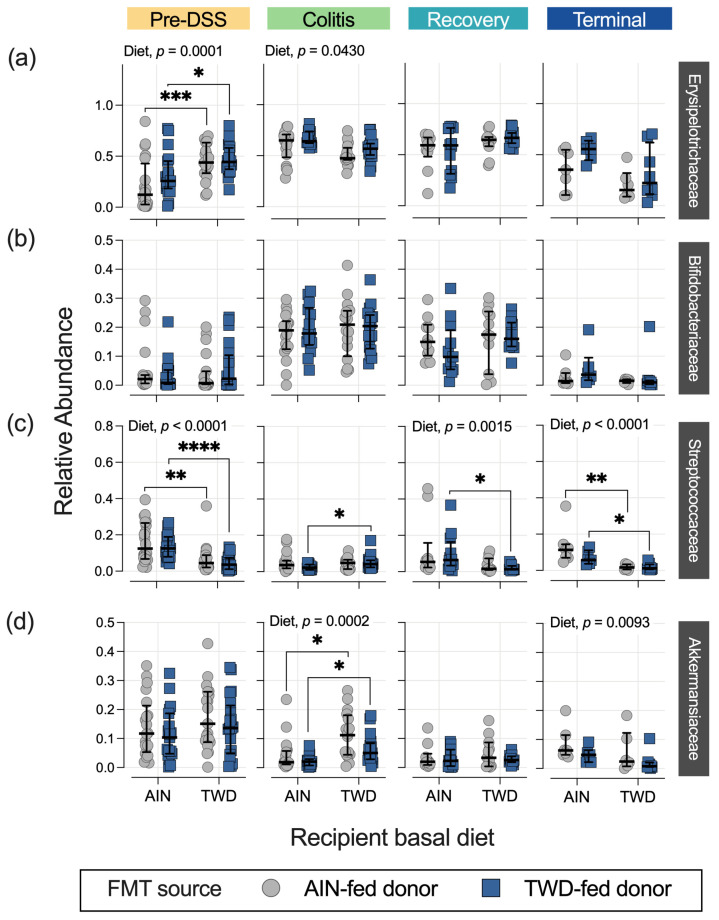
Relative abundance for selected bacterial families of interest at each experimental time point: (**a**) Erysipelotrichaceae, (**b**) Bifidobacteriaceae, (**c**) Streptococcaceae, and (**d**) Akkermansiaceae. The data are shown as individual values that represent each cage (as the biological unit) with median ± interquartile range (*n* = 19 to 20 cages per group at the pre-DSS time point, *n* = 17 to 18 cages per group at the active colitis time point, *n* = 12 to 13 cages per group at the recovery time point, and *n* = 7 to 8 cages per group at the terminal time point). For simplified visualization, this plot shows only the statistical results as FDR-corrected *p*-values for significant main effects or the post hoc comparisons of FMT from the AIN-fed or TWD-fed donors: *, *p* < 0.05; **, *p* < 0.01; ***, *p* < 0.001; and ****, *p* < 0.0001, as outlined in the Materials and Methods section. The complete results of all metagenomeSeq statistical analyses, including pairwise comparisons by recipient basal diet and across time points, are provided in Supplementary File S2.

The taxonomic composition of the fecal microbiome of the recipient mice reflected their basal diet to a much greater extent than the FMT donor source at each experimental stage, although most profoundly at the terminal time point (Figure 4, Figure 5 and Figure S4). It should be noted that the microbiome profiles shown for the donor mice are from the most proximal weekly collection before the indicated study time point for the recipients (i.e., collection 2 for the pre-DSS time point, collection 3 for the colitis time point, collection 5 for the recovery time point, and collection 5 for the terminal time point) (Figure S1). The statistical analyses of microbiome taxonomic abundance via either metagenomeSeq (Figure 5b and Supplementary File S2) or nonparametric heat tree analyses (Figure 6) showed that many more significant differences were detected when comparing the experimental groups according to the basal diet as opposed to comparing them according to the FMT source. For example, at the pre-DSS time point, the relative abundance of Erysipelotrichaceae (*D. newyorkensis*) in the mice fed the TWD diet was significantly greater compared to the AIN-fed mice that received FMT from AIN-fed donors (*p* = 0.0002) or TWD-fed donors (*p* = 0.0153) (Figure 6a and Figure 7a). An overall greater abundance of this bacterial family was also noted during active colitis (diet main effect, *p* = 0.0430), although not at later time point. However, there was no apparent effect of the FMT source on the abundance of Erysipelotrichaceae (Figure 6a and Figure 7a). While changes in the relative abundance of Bifidobacteriaceae were apparent when considering microbiome composition over time, as noted above, our analyses for the other two experimental factors—basal diet and FMT source—did not reveal any significant effects on this bacterial family (Figure 7b).

Significant effects of the basal diet were also apparent for Streptococcaceae (primarily the genus *Lactococcus*) throughout the study, with the relative abundance of this family being significantly higher in the mice fed the AIN diet compared to the mice fed the TWD before the induction of colitis, regardless of the FMT source (*p* < 0.01) (Figure 6a and Figure 7c). By the colitis time point, this trend was altered for those mice that received FMT from TWD-fed donors, with the relative abundance of Streptococcaceae higher in the AIN-fed mice compared to their TWD-fed counterparts (*p* = 0.0272). By the recovery and terminal time points, however, the relative abundance of this family was again similar to the original pre-DSS values, with higher percentages for mice fed the AIN basal diet than for those fed the TWD (diet main effects, *p* = 0.0014 and <0.0001 for recovery and terminal time points, respectively). Again, no significant effects of the FMT source were noted for Streptococcaceae at any point during the study. A similar result was evident for *A. muciniphila* of the Akkermansiaceae family, as no effect of FMT source was apparent. However, the relative abundance of this species increased during active colitis and at the terminal time point for the mice fed the TWD compared to those fed the AIN diet, regardless of the FMT source (Figure 6a and Figure 7d).

**Figure 8:**
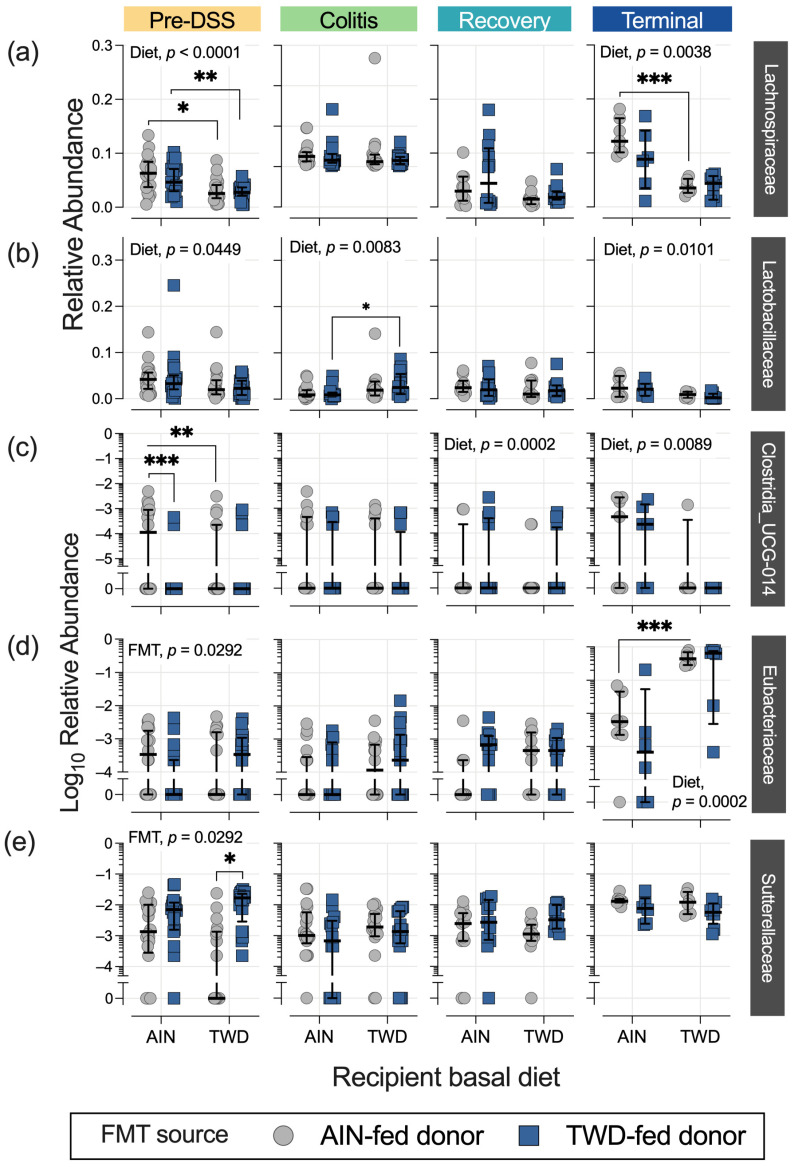
Relative abundance of additional bacterial families of interest at each experimental time point: (**a**) Lachnospiraceae, (**b**) Lactobacillaceae, (**c**) Clostridia_UCG_014, (**d**) Eubacteriaceae, and (**e**) Sutterellaceae. The data are shown as individual values that represent each cage (as the biological unit) with median ± interquartile range (*n* = 19 to 20 cages per group at the pre-DSS time point, *n* = 17 to 18 cages per group at the active colitis time point, *n* = 12 to 13 cages per group at the recovery time point, and *n* = 7 to 8 cages per group at the terminal time point). For simplified visualization, this plot shows only the statistical results as FDR-corrected p-values for significant main effects or the post hoc comparisons of FMT from the AIN-fed or TWD-fed donors: *, *p* < 0.05; **, *p* < 0.01; and ***, *p* < 0.001, as outlined in the Materials and Methods section. The complete results of all metagenomeSeq statistical analyses, including pairwise comparisons by recipient basal diet and across time points, are provided in Supplementary File S2.

The relative abundance of bacteria belonging to the Lachnospiraceae family was higher in the recipient mice fed the AIN diet compared to those provided the TWD, regardless of the FMT donor source, before the induction of colitis at the pre-DSS time point (Figure 6a and Figure 8a). This trend appeared disrupted during active colitis and recovery, with no effect of basal diet observed, but it appeared to be restored at the terminal time point, especially for mice that received FMT from the AIN-fed donor group (*p* = 0.0008). The relative abundance of Lachnospiraceae was not significantly impacted by the FMT source. The relative abundance of Lactobacillaceae also appeared to be reduced in the TWD-fed recipients compared to their AIN-fed counterparts, irrespective of the FMT source, with significant main effects of diet noted prior to the DSS treatment, during active colitis, and at the study end point (Figure 8b). However, as with most other taxa, the FMT source did not alter the relative abundance of this bacterial family either.

FMT from AIN- or TWD-fed donor mice did not alter the relative abundance of most of the taxa identified in this microbiome sequencing study, with a couple of exceptions. Before the onset of colitis, Clostridia_UCG-014 abundance was significantly reduced in the mice that received FMT from the TWD-fed donors (FMT main effect, *p* < 4.25 × 10^−8^), most notably for mice that were fed the AIN basal diet (*p* = 0.0002) (Figure 8c). However, this pattern did not persist throughout the study; instead, significant main effects of basal diet were apparent, regardless of FMT source, with Clostridia_UCG-014 being more abundant in the mice fed the TWD during recovery (diet main effect, *p* = 0.0002) and then less abundant by the terminal time point (*p* = 0.0089). Similarly, the relative abundance of another relatively rare bacterial family, Eubacteriaceae, was higher in mice receiving FMT from the AIN-fed donors, showing a higher abundance at 0.085% of the fecal microbiome compared to their counterparts that received FMT from the TWD-fed donors at 0.056% (FMT main effect, *p* = 0.0292), as observed prior to the DSS treatment (Figure 8d). Conversely, FMT from the TWD-fed donors significantly increased the relative abundance of Sutterellaceae compared to the AIN-fed donors (FMT main effect, *p* = 0.0292), an effect that was most evident in mice provided the TWD (*p* = 0.0153) (Figure 8e). However, these apparent effects of FMT source on the abundance of Eubacteriaceae and Sutterellaceae in the fecal microbiome were transient and did not persist through the colitis, recovery, or terminal time points.

**Figure 9:**
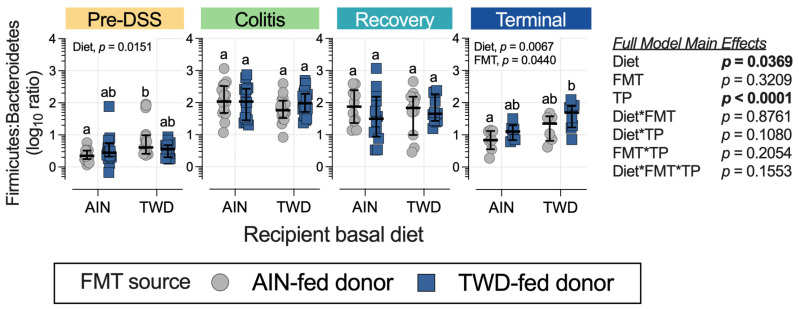
Ratio of Firmicutes to Bacteroidetes at each experimental time point. The ratios were determined using normalized count data for each phylum. The data are shown as individual values representing each cage (as the biological unit) with median ± interquartile range. (*n* = 19 to 20 cages per group at the pre-DSS time point, *n* = 17 to 18 cages per group at the active colitis time point, *n* = 12 to 13 cages per group at the recovery time point, and *n* = 7 to 8 cages per group at the terminal time point). The table shows the statistical model’s main effects, including all experimental factors, and different letters indicate that the experimental groups are significantly different (*p* < 0.05), as determined by the statistical methods outlined in the Materials and Methods section.

Prior to gut insult, the mice fed the TWD as a basal diet had a significantly higher Firmicute-to-Bacteroidetes ratio (F:B) compared to the mice fed the AIN diet, but only for those recipients that received FMT from the AIN-fed donors (Figure 9). The F:B ratio was higher for all groups during the active colitis and recovery time points compared to pre-DSS, although there were no differences in this ratio among the experimental groups. By the end of the study, significant main effects of both diet (*p* = 0.0067) and FMT source (*p* = 0.0440) were observed, with one significant pairwise comparison among the experimental groups for the recipient mice fed the AIN diet with FMT from the AIN-fed donors compared to the recipient mice fed the TWD with FMT from the TWD-fed donors (Figure 9). 

### 3.5. Alpha and Beta Diversity of Fecal Microbiome

**Figure 10:**
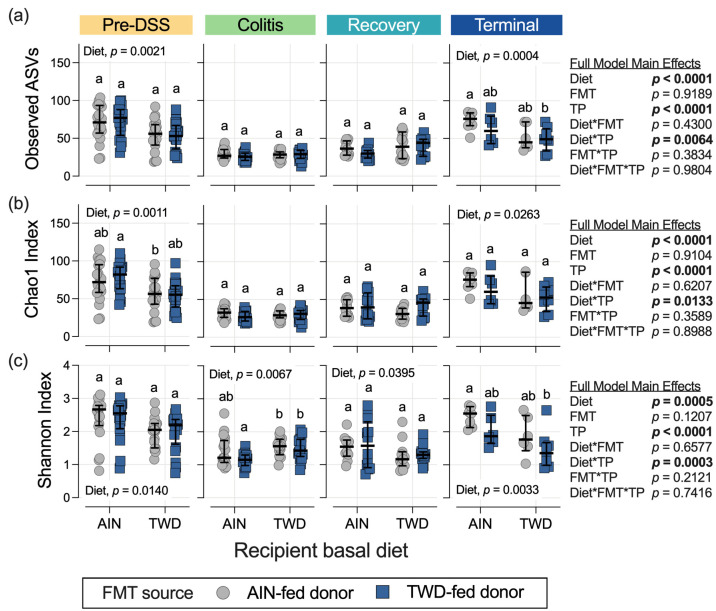
Alpha diversity of mouse fecal microbiomes at each experimental time point. Alpha diversity measures include (**a**) observed ASVs, (**b**) the Chao1 index, and (**c**) the Shannon index. The data are shown as individual values representing each cage (as the biological unit) with median ± interquartile range (*n* = 19 to 20 cages per group at the pre-DSS time point, *n* = 17 to 18 cages per group at the active colitis time point, *n* = 12 to 13 cages per group at the recovery time point, and *n* = 7 to 8 cages per group at the terminal time point). The inserted tables show the statistical model’s main effects, including all experimental factors, for each α-diversity measure. Different letters indicate that the experimental groups are significantly different (*p* < 0.05), as outlined in the Materials and Methods section. Significant main effects of either recipient basal diet or FMT source are also shown. The complete results of these statistical analyses, including all comparisons within and across time points, are found in Tables S2–S4.

The richness and evenness of microbial communities were determined using three indices of alpha diversity measures: observed ASVs (count of sequence variants), Chao1 index (species richness), and Shannon index (community evenness). In a pattern similar to past studies, prior to carcinogen exposure, the alpha diversity was higher in the AIN-fed mice compared to the TWD-fed mice, notwithstanding the FMT source (diet main effect, *p* < 0.05 for all alpha diversity measures) (Figure 10). Compared to the initial alpha diversity measurements, observed ASVs, Chao1, and Shannon indices were markedly lower during the active colitis and recovery phases of this disease model (time point main effect, *p* < 0.0001 for all comparisons) (Table S2). During active colitis, the recipient mice fed the TWD had a higher Shannon index compared to their counterparts fed the AIN basal diet overall (diet main effect, *p* = 0.0067), but this effect was most pronounced when compared to the recipients that were fed the AIN diet and received FMT from the TWD-fed donors (Figure 10c). This pattern was reversed during recovery, as the recipient mice fed the AIN diet had a greater Shannon index compared to the recipient mice fed the TWD (diet main effect, *p* = 0.0395), although none of the pairwise comparisons among the separate experimental groups were significant. Likewise, during the colitis and recovery phases, no differences in the species richness measurements were noted (Figure 10a,b). By the terminal time point, the main effects of diet were apparent for all alpha diversity measurements, with overall higher scores in the recipient mice fed the AIN diet compared to their TWD-fed counterparts. No main effects of FMT source were noted, although observed ASVs and Shannon index was significantly greater in the recipient mice fed the AIN diet and received FMT from the AIN-fed donors when compared to the recipient mice fed the TWD and received FMT from the TWD-fed donors (*p* = 0.0248 and =0.0023 for ASVs and Shannon index, respectively) (Table S3).

**Figure 11:**
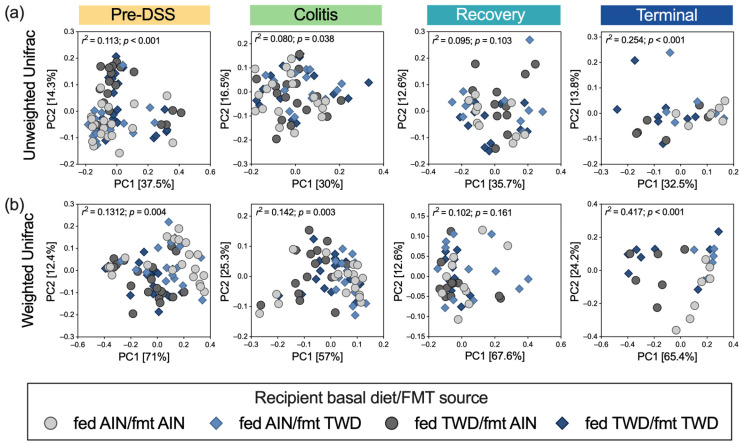
Beta diversity of mouse fecal microbiomes at each experimental time point. The principal coordinate plots depicting fecal microbiome beta diversity using (**a**) unweighted or (**b**) weighted unifrac distances are shown using the first two components (*n* = 19 to 20 cages per group at the pre-DSS time point, *n* = 17 to 18 cages per group at the active colitis time point, *n* = 12 to 13 cages per group at the recovery time point, and *n* = 7 to 8 cages per group at the terminal time point). The variations attributed to PC1 and PC2 are shown, along with the *r^2^* and permanova *p*-values for each plot.

Beta diversity was determined using weighted and unweighted unifrac distance measurements to account for the relative abundance of taxa and the presence of rare taxa, respectively, in the fecal microbiome populations. Before the induction of colitis, clear distinctions in the beta diversity of the fecal microbiome were apparent, with individual samples (corresponding to cages) being grouped more closely according to the diet fed to the recipient mice (permanova *p* < 0.001 for unweighted and *p* = 0.004 for weighted unifrac analyses) (Figure 11). During active colitis, the unweighted unifrac analysis suggested less distinction for these microbiomes, indicating a reduced contribution of rare species during this time point (permanova *p* = 0.038 with low *r*^2^ = 0.08). Alternatively, the weighted analysis suggested some distinction between the recipient mice fed the AIN diet compared to those fed the TWD (permanova *p* = 0.003). During recovery, however, neither beta diversity analysis suggested that the microbiomes were substantially distinct. By the study end point, the microbiomes of the recipient mice fed the AIN diet were distinct from those fed the TWD (permanova *p* < 0.001 for both weighted and unweighted unifrac analyses). Additionally, considering the weighted unifrac analysis at the terminal time point, a moderate separation between the mice that received FMT from the AIN-fed donors and the mice that received FMT from the TWD-fed donors was evident along the second principal coordinate. Otherwise, distinctions among the microbiomes according to the FMT source were not evident at any other time point during the study.

## 4. Discussion

The gut microbiome modulates physiological functions related to cancer development, including inflammation, cell proliferation, apoptosis, and angiogenesis. Thus, it is likely that the gut microbiome directly affects colon tumorigenesis. Patients with IBD or colon tumors have often been observed to have distinct microbiomes, a phenomenon that has also been observed in animal models of these diseases, although a consensus cancer-related gut microbiome has not been identified to date. In this study, we sought to better understand how diet-driven changes in the gut microbiome of donor mice could influence inflammation-associated colorectal carcinogenesis in recipient mice by employing FMT. Importantly, the study design considered the impact of basal diet during disease development for recipient mice that were colonized by fecal microbiomes transferred from mice experiencing either mild or severe colitis and colon tumorigenesis. If our hypothesis that the composition of the gut microbiome is a driving factor in disease development was correct, we would have expected to observe worse colitis symptoms, greater colon tissue inflammation and damage, and a higher tumor incidence in the recipient mice that received FMT from the TWD-fed donors, especially for the recipients that were fed the AIN basal diet, which is known from several previous studies to induce only mild colitis and low tumorigenesis [9]. However, we observed that FMT from either donor had little to no effect on colitis symptoms, colon tissue inflammation, or mucosal injury. Interestingly, the effects of FMT from the TWD-fed donors suggested a potential protective effect against colon tumorigenesis, as reflected by significantly increased colon length and smaller tumor volume as well as trends for reduced tumor multiplicity and volume, although this pattern of response did not align with the observations on gut inflammation as would be expected from our prior work in this CAC model. Importantly, our findings suggest that the basal diet fed to the recipient mice was the major driver of disease progression in this experiment. Likewise, the composition of the fecal microbiomes of the recipient mice was also affected to a much greater extent by the diet they consumed as opposed to the source of FMT.

FMT is a useful approach for shifting the gut microbiome population in favor of health-promoting bacteria and, potentially, reversing gut dysbiosis [28]. Previously, FMT was shown to be successful in the treatment of *Clostridium difficile* infection [29]. However, in 2019, the U.S. Food and Drug Administration warned of a high transmission risk of pathogenic, drug-resistant bacteria following FMT intended to treat *C. difficile* infection [30]. As reviewed recently by Waller et al. [31], the evidence for the use of FMT to induce remission in patients with mild-to-moderate IBD is substantial, as multiple randomized, controlled trials have yielded positive results after 8 to 12 weeks and noted benefit compared to placebo. While FMT from a healthy microbiome appears to assist in disease management, FMT from an unhealthy source may potentially lead to unfavorable outcomes.

As observed repeatedly in this murine model of CAC [9,22,32], mice fed the TWD (irrespective of FMT source) had shorter colon length, more severe colitis symptoms, higher inflammation and mucosa injury scores, higher tumor multiplicity, larger average tumor volume, and higher tumor burden compared to their AIN-fed counterparts. Interestingly, in this experiment, we also observed a marked increase in colon tumor incidence in the TWD-fed recipient mice. Furthermore, the mice fed the TWD consumed the same amount of food as the mice fed the AIN diet, resulting in increased energy consumption in the mice fed the TWD due to its higher energy density. However, final body weight and body composition were not significantly affected. In summary, in this study, the diet fed to the recipient mice was the main driver for inflammation response during active colitis, recovery from gut injury, and tumorigenesis. Interestingly, we arrived at a similar conclusion in a prior study using donor fecal material from obese and lean human donors transferred to recipient mice fed different basal diets, including the AIN93G diet and TWD [21]; in that human-to-mouse FMT experiment, the basal diet fed to the recipient mice was the primary driver for the development of metabolic syndrome and obesity.

However, a few outcomes of this study were significantly affected by the FMT source, namely AIN-fed versus TWD-fed donor mice. Of note, while the inflammation score at the recovery time point and the ratio of Firmicutes: Bacteroidetes at the terminal time point increased with FMT from the TWD-fed donors, an improvement in colon length and average tumor volume was observed in the recipient mice that received FMT from the TWD-fed donors compared to those that received FMT from the AIN-fed donors, irrespective of the basal diet they consumed. However, an explanation for the contradictory pattern in these outcomes is not entirely straightforward. First, greater colon length is generally associated with reduced inflammation and a lower Firmicutes: Bacteroidetes ratio [9,33,34]. Furthermore, the higher inflammation score observed during recovery for the mice that received FMT from the TWD-fed donors compared to the mice that received FMT from the AIN-fed donors (significant main effect of FMT source) suggests that the microbiome associated with the TWD might delay recovery from gut injury. However, these higher inflammation scores during recovery do not correlate with the observed lower average tumor multiplicity, average tumor volume, and tumor burden measures at the terminal time point for those mice that received FMT from the TWD-fed donors. Rather, based on our prior observations [9], we would have expected more severe tumorigenesis in mice that experienced prolonged inflammation through the recovery period. Additionally, the evidence for an association of a higher abundance of Firmicutes in the fecal microbiome with IBD and CRC is equivocal [35,36,37,38,39]. For example, in a metagenomic analysis of fecal samples obtained from 290 healthy subjects, 512 IBD patients, and 285 CRC patients, Ma et al. reported that IBD patients had low microbial diversity, whereas diversity was elevated in CRC patients. Furthermore, they found that the ratio of Firmicutes: Bacteroidetes was lower in these patients compared to their healthy counterparts [40]. This pattern differs from our findings, which showed that the overall Firmicutes: Bacteroidetes ratio was elevated during colitis; furthermore, the ratio was also elevated in the mice that experienced more severe tumorigenesis at the study end point (i.e., those recipient mice that were fed the TWD).

The application of FMT to ameliorate adverse side effects, such as mucosal inflammation, associated with some common chemotherapies used to treat CRC in humans has gained interest recently. For example, Chang et al. showed that FMT from healthy mice reduced mucosal inflammation and improved gut barrier integrity in Balb/c mice implanted with syngeneic CT26 colon adenocarcinoma cells on a FOLFOX regimen [41]. Additionally, vancomycin-pretreated Balb/c mice administered 5-fluorouracil to induce mucositis were treated with FMT from a pool of healthy mice donors, resulting in the prevention of weight loss and colon shortening [42]. Finally, in a CAC model employing AOM with three cycles of DSS, female Balb/c mice were administered oral FMT from healthy, age- and sex-matched donors following DSS treatments, leading to less severe disease response as indicated by longer colon length, reduced gut inflammation, and reduced tumor burden, compared to mice that did not receive FMT [43]. Of note, none of these studies considered the role of basal diet given to recipient mice in mitigating or accentuating the effects of FMT on colitis or colon carcinogenesis. Furthermore, FMT was typically administered in combination with or following intense gut injury caused by chemical exposure, including treatments that likely depleted the resident gut microbiome, allowing for more efficient colonization by bacteria through FMT.

In this study, FMT from the donor mice fed either the AIN diet or the TWD diet did not significantly affect the richness or evenness of the fecal microbiome of the recipient mice. Likewise, neither weighted nor unweighted unifrac β-diversity analyses suggested that the populations of bacteria in the stool samples of the recipient mice were different according to the FMT source. However, we did observe that FMT from the donor mice fed the TWD, before DSS-induced colitis, reduced the relative abundance of two bacterial families, Clostridia_UCG-014 and Eubacteriaceae, both in the order Clostridia. In a study involving patients diagnosed with Crohn’s disease and a cohort of healthy first-degree relatives, Leibovitzh et al. determined that the abundance of Clostridia_UCG-014 was associated with impaired intestinal permeability, although this association appeared to be independent of gut inflammation [44]. A recent fecal microbiota analysis of UC patients indicated a reduced abundance of *Clostridium cluster IV* and *Eubacterium rectale* compared to healthy controls [45]. Of note, in the current study, the overall abundance of Clostridia_UCG-014 was not significantly different in the mice experiencing colitis compared to the pre-DSS baseline measurement, although reduced abundance was evident in the mice recovering from colitis and at the study end point. Alternatively, the relative abundance of Eubacteriaceae was not altered during active colitis or recovery, but it was notably elevated by the terminal time point. Species belonging to the Eubacteriaceae family have demonstrated anti-inflammatory properties and have been reported in reduced amounts in animal models of CAC and IBD patients [46,47]. Eubacteria species are butyrate producers, which are the main source of colonocyte energy, potentially explaining the increase in this species in mice with significant tumor development [48].

Another bacterial species notably affected by FMT from the TWD-fed donor mice before the induction of colitis was *Parasutterella uncultured_organism* (family Sutterellaceae); furthermore, this species was substantially more abundant at the terminal time point compared to all prior time points, regardless of the experimental group. In a cross-sectional study of an Italian cohort of IBD patients, the researchers determined that *Sutterella spp*. was elevated in the stool of IBD patients compared to healthy controls [49]. However, as noted by Kaakoush [50], while a high abundance of *Sutterella* has been associated with UC, evidence suggests that this species does not induce gut inflammation, but rather it degrades gut-associated immunoglobin A, which is essential for protection against bacterial invasion [50]. Given the strong association of *Sutterella* with colitis, the observation that the mice that received FMT from the TWD-fed donors harbored a greater abundance of *Parasutterella* before colitis induction, and the functional similarity of both *Sutterella* and *Parasutterella* [51], it would be reasonable to expect that FMT from the TWD-fed mice might lead to more severe colitis in the recipient mice, especially those recipients fed the AIN diet, for which a mild inflammatory response would otherwise be expected without any intervention. However, this was not the case in our study, and no further effect of FMT on *Parasutterella* abundance was noted throughout the experiment.

In this study, the basal diet fed to the recipient mice had much more profound effects on the fecal microbiomes of the recipient mice than did FMT from the donor mice fed either the AIN diet or the TWD. Prior to the induction of colitis, α-diversity was significantly higher in the mice fed the standard AIN diet when compared to their TWD-fed counterparts. While species richness was generally low for all experimental groups during active colitis and recovery from gut injury, this trend returned by the end of the study, with the AIN-fed mice having more taxa present in their fecal microbiomes than the mice provided the TWD. A similar pattern for beta diversity was also evident, particularly when considering the most abundant species in the fecal microbiomes of the recipient mice; the microbiomes of the mice provided the AIN diet, irrespective of FMT source, were notably distinct from their TWD-fed counterparts before the onset of colitis and at the study end point. The negative impact of the Western dietary pattern on gut microbiome diversity compared to healthier diets, such as those high in fiber and polyunsaturated fatty acids similar to the Mediterranean or Japanese diets, has been well established [52,53,54,55].

*A. muciniphila* is a highly studied anaerobic bacterium that resides within the mucosal layer of the intestinal mucosa; this mucin-degrading bacterium is thought to contribute to intestinal homeostasis and gut health [56,57,58]. However, the role of A. muciniphila in the development of gastrointestinal disease is not yet well understood. Some studies have reported an elevated abundance of this bacterium in CRC patients [59,60], whereas others have reported reduced abundance in patients diagnosed with IBD [45,61,62,63]. Håkansson and colleagues measured a higher abundance of *A. muciniphila* in the colon mucosal tissue of mice with DSS-induced colitis compared to non-treated controls [64], a finding that does not agree with the overall lower abundance of this bacterium during DSS-induced colitis in this study and our prior work [9], although the experimental protocols were notably different (10 mg/kg AOM + 1% DSS for 10 days in this study vs. 4% DSS for 7 days in Håkansson et al.). Of interest, Qu et al. reported that oral administration of *A. muciniphila* ameliorated symptoms of DSS-induced colitis in mice [65]. Collectively, these data point to a complicated role of *A. muciniphila* in gut homeostasis and disease development.

Several of the taxa that demonstrated dynamic relative abundance throughout disease development in response to the basal diet have also been implicated in IBD and/or CRC. We determined that members of the Lachnospiraceae family (genera *Lachnospiraceae_NK4A136_group*, *Lachnospiraceae_A2*, *Blautia*, *Marvinbryantia,* and *Lachnoclostridium*) were less abundant in the TWD-fed recipient mice compared to their AIN-fed counterparts prior to gut injury, and at the study end point in mice with high tumor burden. Several studies have reported reduced levels of *Blautia spp.* in the fecal microbiome of CRC patients compared to healthy controls [66,67,68]. Wang et al. reported a decrease in the abundance of Lachnoclostridium sp. in both wild-type and TGFβ-deficient mice after the induction of colorectal carcinogenesis by AOM + DSS [69], a finding that appears to differ from our observations for the Lachnospiraceae family, including *Lachnoclostridium spp*.; however, we do note that the abundance of this taxa is reduced at the study end point in mice fed the TWD, which have much more severe tumor outcomes.

Similar to the findings reported in our prior study [22], we observed in this experiment that the relative abundances of bacteria in the Erysipelotrichaceae (e.g., *Turicibacter* spp. and *D. newyorkensis*) and Bifidobacteriaceae (*Bifidobacterium* spp.) families were elevated in the fecal microbiomes of mice experiencing active colitis compared to their pre-DSS counterparts. A greater abundance of Erysipelotrichaceae in the fecal microbiome has been found in the lumen of CRC patients compared to a healthy control group and to other gut diseases [70,71]. Additionally, in a murine colitis model, consumption of a choline-deficient diet led to a decrease in relative abundance of Erysipelotrichaceae [72]. Moreover, *Bifidobacterium* appears to increase in abundance during active IBD and has been reported as a predominant genus in the microbiome of Taiwanese IBD patients [73,74,75,76]. On the other hand, administration of *Bifidobacterium longum* suppressed development of preneoplastic lesions in mice and appeared to induce expression of tumor-suppressing microRNAs [77], whereas *Bifidobacterium infantis* conferred protection against DSS-induced colitis and abnormal immune signaling [77,78]. Additionally, various strains of *Bifidobacterium* have anti-cancer properties and have been used as probiotics [79,80].

This study had several limitations to consider. Although a universal protocol for FMT has not yet been established, researchers have determined that FMT into recipients with a depleted gut microbiome—either via the use of antibiotic or polyethylene glycol administration or via the use of germ-free organisms—is more successful than FMT into recipients with intact gut microbiomes [81]. In this experiment, broad-scope antibiotics were used to deplete the resident microbiome of the recipient mice prior to FMT and implementation of the AOM/DSS to induce colitis and colon tumorigenesis. However, with this method, it is possible that some microbiota persisted, and antibiotic use could select for resistant bacteria or allow the overgrowth of other microbes. These drawbacks could be avoided by using germ-free mice [82]. However, germ-free mice present other disadvantages, and chief among them is an improperly developed gut immune system. Furthermore, FMT does not provide a perfect transplantation of donor microbiome to the gut of the recipient. Some species are unable to successfully colonize the recipient’s gut, although this issue is less of a concern in the present study, which employed mouse-to-mouse FMT, compared to human-to-mouse FMT studies. Furthermore, the collection and storage methods used in this study might not have preserved all bacterial species, such as obligate anaerobes [81]. Additionally, the 16s rRNA sequencing analyses of fecal microbiomes generated the data for the relative abundance of bacteria, not their numerical abundance in the experimental samples. Thus, apparent changes in relative abundance of a particular taxon should not be interpreted as a change in its actual population size; rather, it is possible that the relative abundance could reflect a growth or a loss of other bacteria in the fecal microbiome community. Another limitation of this work was the narrow focus of the experimental design, which centered on the question of whether the gut microbiome from mice experiencing Western diet-enhanced colitis and colon tumorigenesis could exacerbate disease in recipient mice that would otherwise experience mild symptoms (e.g., the AIN-fed recipients). This study’s design did not address the question of whether the gut microbiome of mice with diet-driven severe disease would induce disease in otherwise healthy mice, which was beyond the scope of this work. Finally, this murine model of CAC allows the generation of colon adenocarcinomas within about six months of tumor initiation by AOM + DSS; however, this model is not generally used to assess the effects of diet or the influence of the gut microbiome on progression to advanced metastatic disease as the rapid growth of colon polyps often requires humane euthanasia of experimental animals due to bowel obstruction.

## 5. Conclusions

In conclusion, the findings of the present study did not support our hypothesis that FMT from mice fed a Western diet, which enhanced colitis and colon tumorigenesis in donor mice, would exacerbate disease symptoms in recipients fed the standard AIN diet. FMT from the donor mice that were fed the TWD and had a severe disease phenotype led to only minor changes in the recipient fecal microbiome and did not worsen disease symptoms in the mice fed the AIN diet. Conversely, FMT from the healthy mice with only mild colitis symptoms did not confer protection against gut inflammation or dysbiosis in the recipient mice fed the TWD. Instead, we determined that the basal diet fed to the recipient mice had a much more substantial effect on colitis, colon tumorigenesis, and fecal microbiome profile of recipient mice, a finding that is consistent with prior work by our group that employed FMT from obese or lean human donors to mice fed differing basal diets [21]. Collectively, these findings point to the need to appropriately consider the influence of basal diet on a recipient’s microbiome in future pre-clinical FMT experiments. Additionally, these observations suggest that the nutritional status of a patient and their routine dietary intakes must be considered when employing FMT as a therapeutic approach for ameliorating gut inflammation. Lastly, future pre-clinical studies may consider whether gut microbiome associated with chronic intake of a Western type of diet may contribute to gut inflammation and/or development of colorectal cancer in otherwise healthy mice by employing a long-term, repeated FMT protocol without the AOM + DSS protocol to initiate CAC.

## Data Availability

Supporting sequencing data for this manuscript are available to the public at the Utah State University Digital Commons repository, https://doi.org/10.26078/z54v-8j64 (accessed on 30 January 2023). Available files include the .txt mapping file with sample attribute information, the .csv file with 16S rRNA sequence count data with ASV identifiers, and the .csv file with taxonomy mapped to the ASV identifiers. All other data are contained within the article and the accompanying Supplementary Material.

## Supplementary Materials

The following supporting information can be downloaded at https://www.mdpi.com/article/10.3390/nu15061338/s1. Figure S1: Experimental design; Figure S2: Relative liver, kidney, spleen, and cecum content weights; Figure S3: Rarefaction curve analysis by experimental group and time point; Figure S4: Taxonomic classification of mouse fecal bacteria at the phylum level; Figure S5: Relative abundance of selected bacterial families of interest across study time points; Table S1: Experimental diet formulations; Table S2: Alpha diversity pairwise comparisons for the effects of time point only, irrespective of basal diet or FMT source; Table S3: Alpha diversity pairwise comparisons by experimental group within time points; Table S4: Alpha diversity pairwise comparisons by time point within experimental group; File S1. Microbiome count data.xlsx; File S2. MetagenomeSeq statistics.xlsx.
