# Supplementary material for: Basal Diet Fed to Recipient Mice Was the Driving Factor for Colitis and Colon Tumorigenesis, despite Fecal Microbiota Transfer from Mice with Severe or Mild Disease
Source: Nutrients. 2023 Mar 9;15(6):1338. doi: 10.3390/nu15061338 (PMC10052649; doi:10.3390/nu15061338)
Supplement: Supplementary file 1 [file nutrients-15-01338-s001.zip › Supplementary Material_revised.pdf]

nutrients

MDPI

Supplementary Material

Basal diet fed to recipient mice was the driving factor for colitis and colon tumorigenesis, despite fecal microbiota transfer from mice with severe or mild disease

Daphne M. Rodriguez, Korry J. Hintze, Giovanni Rompato, Eliza C. Stewart, Abbey H. Barton, Emily Mortensen-Curtis, Porter A. Green, Arnaud J. Van Wettere, Aaron J. Thomas, Abby D. Benninghoff *

* Correspondence: abby.benninghoff@usu.edu; Tel.: +01-435-797-8649

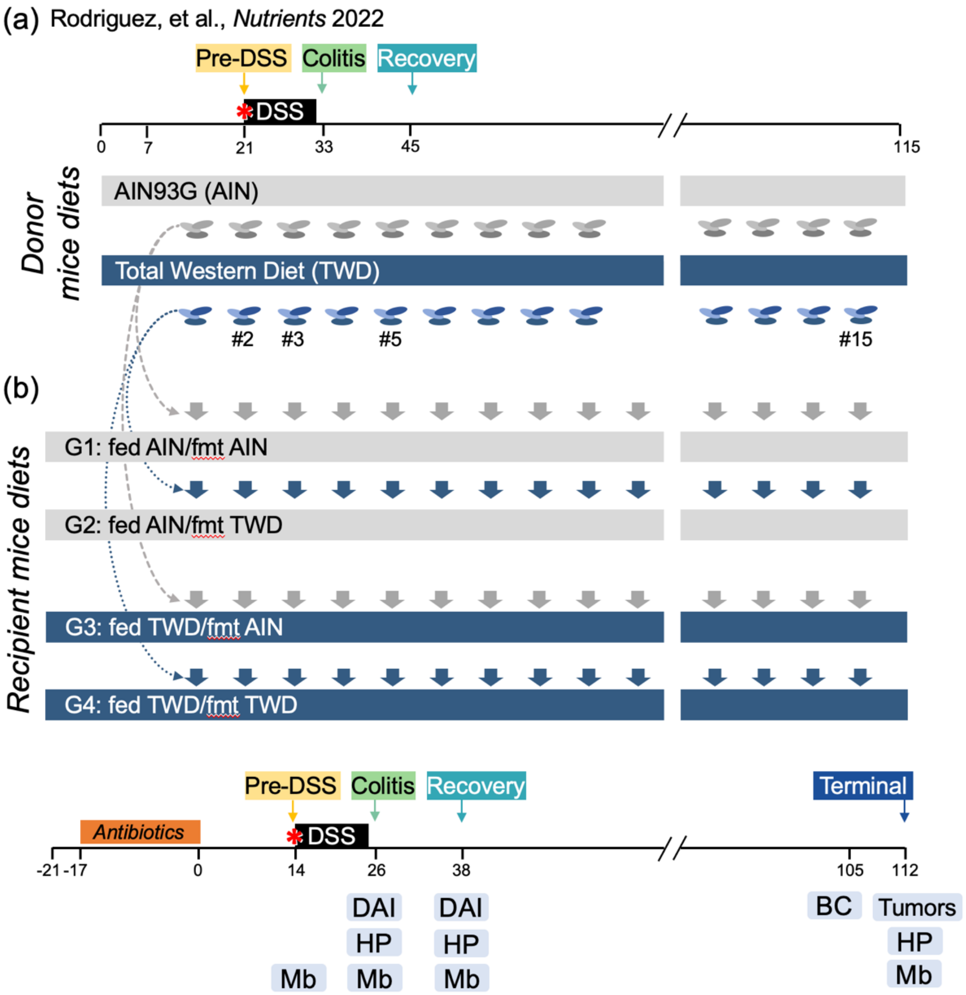

Figure S1. Experimental design. (**a**) Diagram outlines the experimental design for the treatment of donor mice with azoxymethane (red asterisk) and dextran sodium sulfate (DSS) to induce colitis and colon tumorigenesis. Also shown are the time points for weekly collection of fecal samples for each basal diet group. Full experimental details are outlined in Rodriguez, et al. [22]. (**b**) Diagram outlines the experiment design for this study, described in detail in the Materials and Methods. Basal diets are represented as gray (AIN) or blue (TWD) bars with FMT from AIN-fed donors (grey arrows) and FMT from TWD-fed donor mice (blue arrows). Numbers below each FMT donor represents the week of collection that was then to time-matched for recipient mice. For example, collection 3 occurred while the donor mice were experience active colitis and was used to inoculate recipient mice just prior to DSS treatment. In this study, the endpoints assessed are shown, including the disease activity index (DAI), histopathology (HP), fecal microbiome profile (Mb), body composition (BC) and colon tumor incidence, multiplicity and burden (tumors).

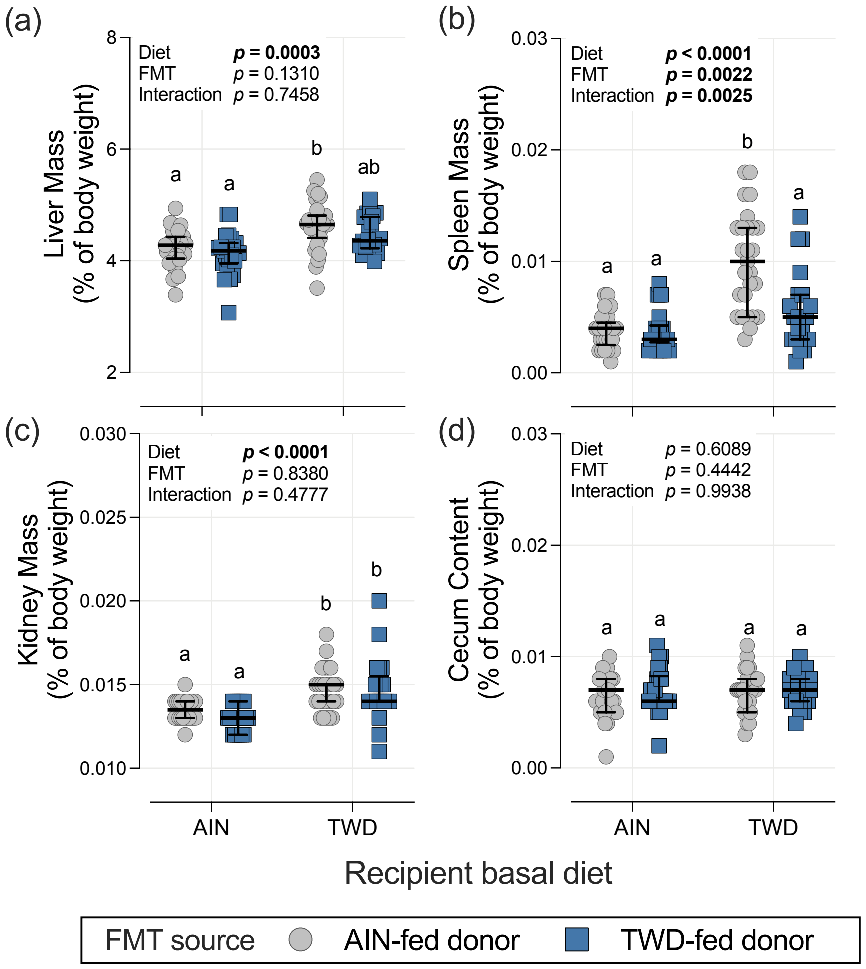

Figure S2. Relative liver, kidney, spleen, and cecum content weights. Data for liver (**a**), kidney (**b**), spleen (**c**), and cecum content (**d**) weights are shown as a proportion of the final body weight. Values for individual mice are shown with median ± interquartile range. Inset tables provide the model main effects for diet, treatment, and their interaction, and different letters indicate groups are significantly different ($p < 0.05$) as outlined in Materials and Methods.

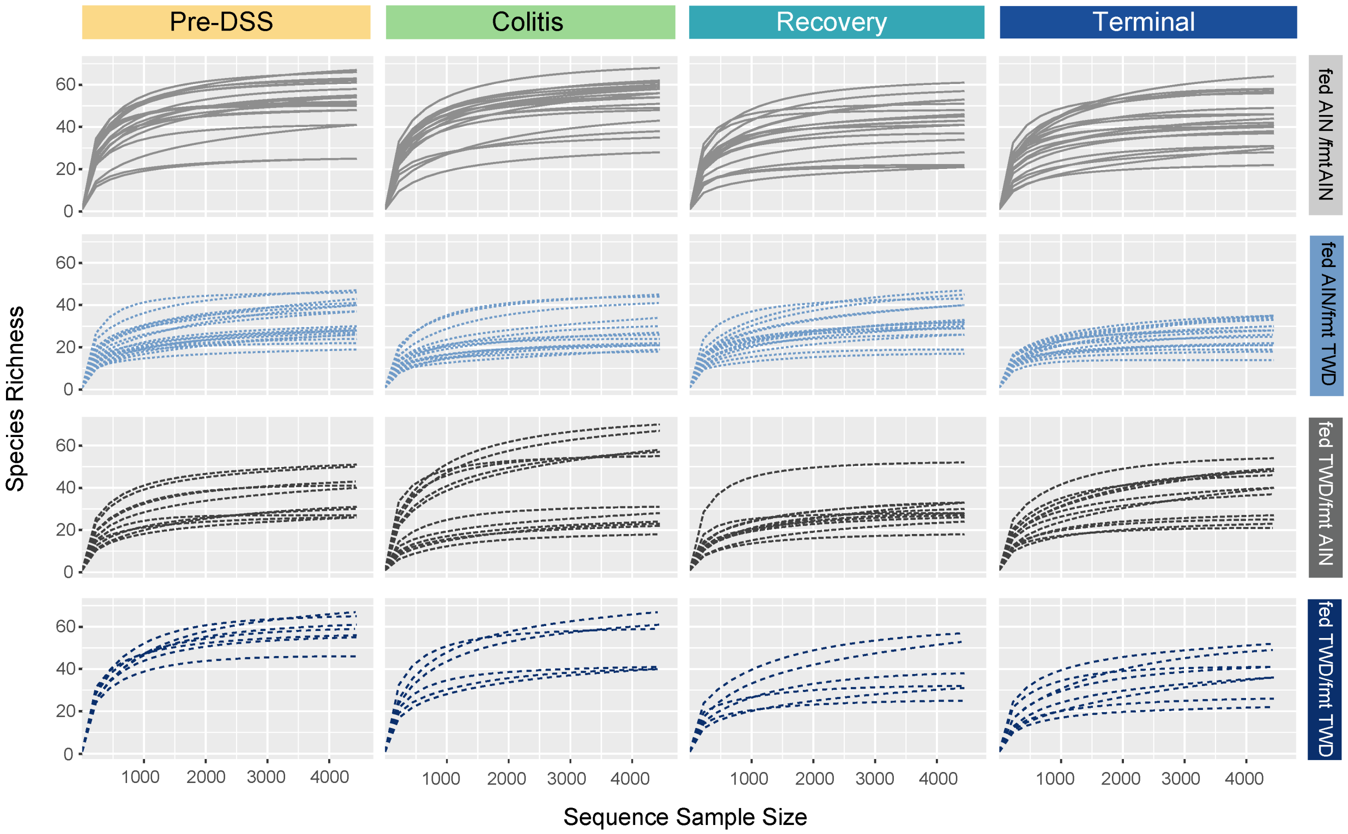

Figure S3. Rarefaction curve analysis by experimental group and time point. Rarefaction curve analysis by experimental group and time point. Curves plot species richness as a function of sequence sample size. For comparisons across experimental groups, data were rarefied to ~4,400 sequences, the lowest total among all the samples. These curves indicate that saturation was reached satisfactorily for most samples and that additional sequence reads were unlikely to substantially increase the number of species detected.

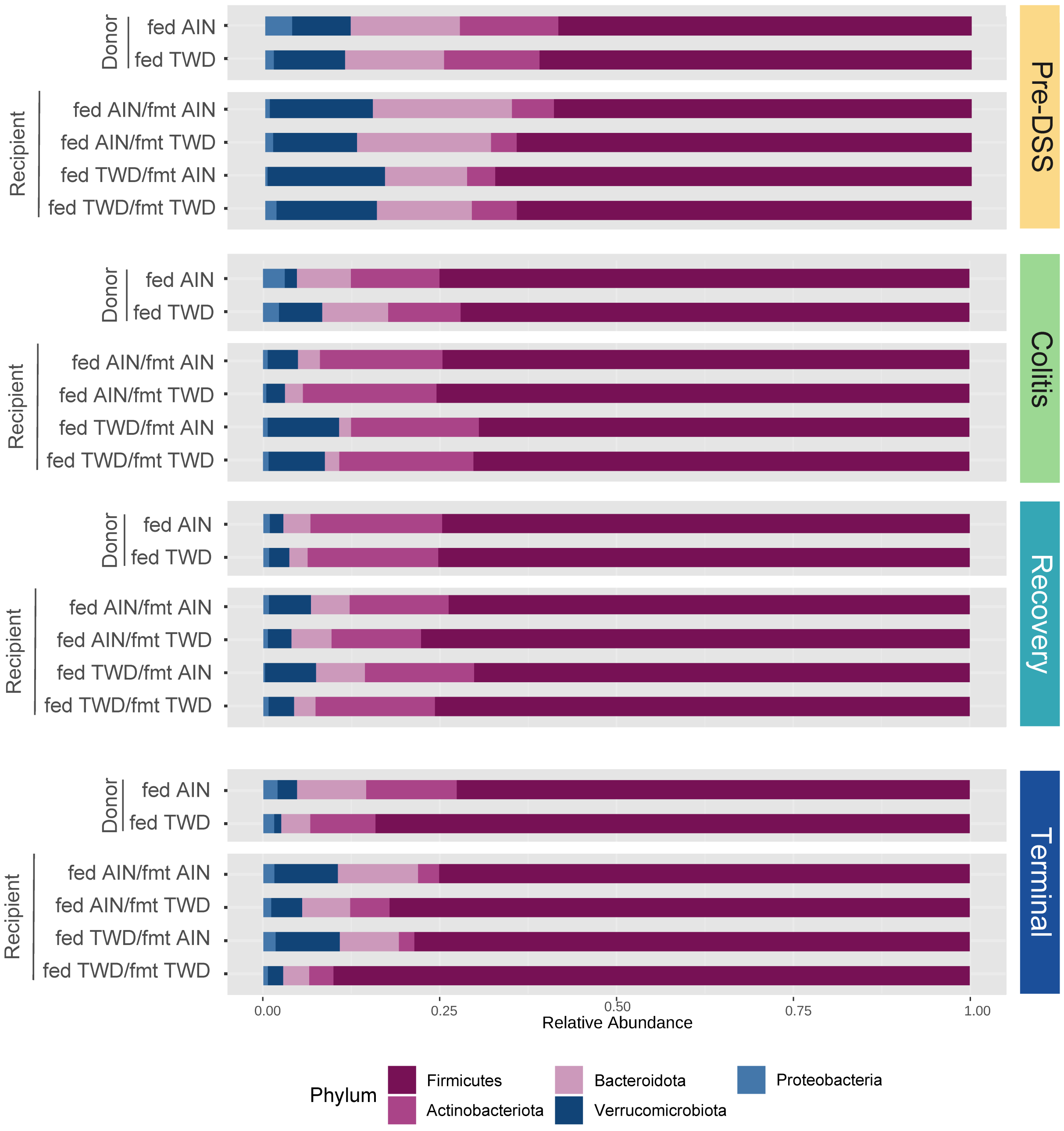

Figure S4. Taxonomic classification of mouse fecal bacteria at the phylum level. Data shown are the relative normalized abundance of bacteria annotated to the phylum level.

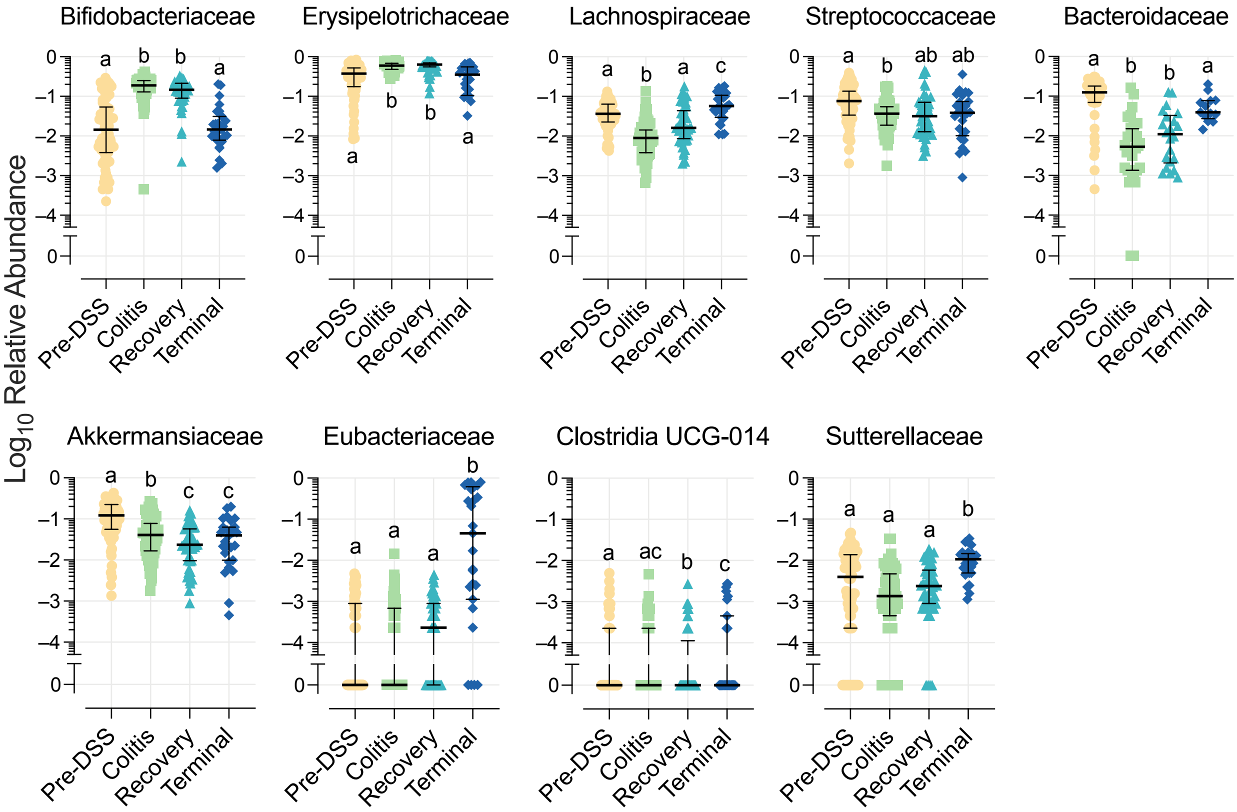

Figure S5. Relative abundance of selected bacteria families of interest over the study time points. The $\log_{10}$ relative abundance values for representing each cage (as the biological unit) with the median ± interquartile range are shown for selected taxa, irrespective of basal diet or FMT donor source, for each experimental time point. Different letters indicate that relative abundances for time points are significantly different ($p < 0.05$) as determined by statistical methods outlined in Materials and Methods.

Table S1. Experimental diet formulations

			AIN93G	TWD
Energy density (kcal/g)			3.76	4.35
Macronutrients	Carbohydrates (g/kg diet)			
		Corn Starch	397.5	230
		Maltodextrin	132	70
		Sucrose	100	261.2
		Cellulose	50	30
	Kcal (% of total)		63.9%	50.0%
	Proteins (g/kg)	Casein	200	190
		L-cystine	3	2.85
	Kcal (% of total)		18.8%	15.5%
	Fats (g/kg)	Soybean oil	70	31.4
		Anhydrous milk fat		36.3
		Olive Oil		28
		Lard		28
		Beef tallow		24.8
		Corn oil		16.5
		Cholesterol		0.4
	Kcal (% of total)		17.2%	34.5%
	Minerals (mg/kg)	Calcium	5000	2011
Micronutrients		Phosphorus	3000	2757
		Sodium	1019	7078
		Potassium	3600	5333
		Magnesium	507	589
		Iron	35	31
		Zinc	30	25
		Copper	6	2.6
		Selenium	0.15	0.2
	Vitamins (unit/kg)	Thiamin (mg)	5	3.5
		Riboflavin (mg)	6	4.4
		Niacin (mg)	30	50.6
		Pyridoxine (mg)	6	3.9
		Folate (mg)	2	1.3
		Vitamin B12 (µg)	25	11
		Vitamin A (IU)	4000	4300
		Vitamin D (IU)	1000	391
		Vitamin E (IU)	75	24.6
		Vitamin K (µg)	750	189
		Choline (mg)	1027	648

Note: TWD, total Western diet. Composition of the TWD was published previously.[1] No data are available in NHANES for chloride, manganese, iodine, pantothenic acid, biotin, or ultra-trace minerals.

[1] Hintze, K.J.; Benninghoff, A.D.; Ward, R.E. Formulation of the total western diet (TWD) as a basal diet for rodent cancer studies. J. Agric. Food Chem. 2012, 60, 6736-6742, doi:10.1021/jf204509a.

Table S2. Alpha diversity pairwise comparisons for effects of time point only, irrespective of basal diet or FMT source

	Alpha Diversity Measure		
**Time point comparison**	**Observed ASVs**	**Chao1 index**	**Shannon Index**
Pre-DSS vs. Colitis	**0.0001**	**0.0001**	**0.0001**
Pre-DSS vs. Recovery	**0.0001**	**0.0001**	**0.0001**
Pre-DSS vs. Terminal	0.7358	0.7080	0.1370
Colitis vs. Recovery	**0.0235**	**0.0444**	0.9776
Colitis vs. Terminal	**0.0001**	**0.0001**	**0.0001**
Recovery vs. Terminal	**0.0001**	**0.0001**	**0.0005**

Values shown are the Tukey HSD post-hoc comparison p-values for each pairwise comparison between experimental diet groups within each timepoint following a generalized linear model analysis. Main effects of all experimental factors are presented in Figure 10.

Table S3. Alpha diversity pairwise comparisons by experimental group within time points

		Alpha Diversity Measure		
Timepoint	Comparison	Observed ASVs	Chao1 index	Shannon Index
Pre-DSS	fed AIN/fmt AIN vs. fed AIN/fmt TWD	0.9999	0.9646	0.9298
Pre-DSS	fed AIN/fmt AIN vs. fed TWD/fmt AIN	0.0971	0.1128	0.0876
Pre-DSS	fed AIN/fmt AIN vs. fed TWD/fmt TWD	0.1385	0.1578	0.2483
Pre-DSS	fed AIN/fmt TWD vs. fed TWD/fmt AIN	0.1026	**0.0470**	0.3331
Pre-DSS	fed AIN/fmt TWD vs. fed TWD/fmt TWD	0.1450	0.0669	0.6455
Pre-DSS	fed TWD/fmt AIN vs. fed TWD/fmt TWD	0.9945	0.9950	0.9324
Colitis	fed AIN/fmt AIN vs. fed AIN/fmt TWD	0.2367	0.3275	0.1366
Colitis	fed AIN/fmt AIN vs. fed TWD/fmt AIN	0.7934	0.6447	0.6840
Colitis	fed AIN/fmt AIN vs. fed TWD/fmt TWD	0.7091	0.6474	0.8974
Colitis	fed AIN/fmt TWD vs. fed TWD/fmt AIN	0.7558	0.9430	**0.0121**
Colitis	fed AIN/fmt TWD vs. fed TWD/fmt TWD	0.8159	0.9344	**0.0320**
Colitis	fed TWD/fmt AIN vs. fed TWD/fmt TWD	0.9992	0.9999	0.9752
Recovery	fed AIN/fmt AIN vs. fed AIN/fmt TWD	0.8925	0.9361	0.9176
Recovery	fed AIN/fmt AIN vs. fed TWD/fmt AIN	0.4851	0.4855	0.6247
Recovery	fed AIN/fmt AIN vs. fed TWD/fmt TWD	0.9771	0.9831	0.6889
Recovery	fed AIN/fmt TWD vs. fed TWD/fmt AIN	0.1407	0.1802	0.2450
Recovery	fed AIN/fmt TWD vs. fed TWD/fmt TWD	0.9862	0.9945	0.2792
Recovery	fed TWD/fmt AIN vs. fed TWD/fmt TWD	0.2247	0.2418	0.9986
Terminal	fed AIN/fmt AIN vs. fed AIN/fmt TWD	0.8964	0.9356	0.6431
Terminal	fed AIN/fmt AIN vs. fed TWD/fmt AIN	0.0916	0.3458	0.1600
Terminal	fed AIN/fmt AIN vs. fed TWD/fmt TWD	**0.0248**	0.0841	**0.0023**
Terminal	fed AIN/fmt TWD vs. fed TWD/fmt AIN	0.4012	0.7522	0.8278
Terminal	fed AIN/fmt TWD vs. fed TWD/fmt TWD	0.1969	0.3678	0.0944
Terminal	fed TWD/fmt AIN vs. fed TWD/fmt TWD	0.9855	0.9284	0.3889

Values shown are the Tukey HSD post-hoc comparison *p*-values for each pairwise comparison between experimental diet groups within each timepoint following a generalized linear model analysis.

Table S4. Alpha diversity pairwise comparisons by time point within experimental group

		Alpha Diversity Measure		
Diet/Treatment	Comparison	Observed ASVs	Chao1 index	Shannon index
fed AIN/fmt AIN	Pre-DSS vs. Colitis	**0.0001**	**0.0001**	**0.0001**
fed AIN/fmt AIN	Pre-DSS vs. Recovery	**0.0001**	**0.0001**	**0.0003**
fed AIN/fmt AIN	Pre-DSS vs. Terminal	0.9860	0.9989	0.9961
fed AIN/fmt AIN	Colitis vs. Recovery	0.6488	0.7368	0.9490
fed AIN/fmt AIN	Colitis vs. Terminal	**0.0001**	**0.0001**	**0.0002**
fed AIN/fmt AIN	Recovery vs. Terminal	**0.0003**	**0.0012**	**0.0031**
fed AIN/fmt TWD	Pre-DSS vs. Colitis	**0.0001**	**0.0001**	**0.0001**
fed AIN/fmt TWD	Pre-DSS vs. Recovery	**0.0003**	**0.0001**	**0.0207**
fed AIN/fmt TWD	Pre-DSS vs. Terminal	0.9547	0.7479	0.9239
fed AIN/fmt TWD	Colitis vs. Recovery	0.1196	0.1449	0.0986
fed AIN/fmt TWD	Colitis vs. Terminal	**0.0002**	**0.0003**	**0.0063**
fed AIN/fmt TWD	Recovery vs. Terminal	**0.0415**	**0.0413**	0.3971
fed TWD/fmt AIN	Pre-DSS vs. Colitis	**0.0001**	**0.0001**	**0.0263**
fed TWD/fmt AIN	Pre-DSS vs. Recovery	**0.0003**	**0.0013**	**0.0004**
fed TWD/fmt AIN	Pre-DSS vs. Terminal	0.9728	0.9999	0.9635
fed TWD/fmt AIN	Colitis vs. Recovery	0.9896	0.9914	0.3445
fed TWD/fmt AIN	Colitis vs. Terminal	**0.0085**	**0.0067**	0.3866
fed TWD/fmt AIN	Recovery vs. Terminal	**0.0268**	**0.0211**	**0.0349**
fed TWD/fmt TWD	Pre-DSS vs. Colitis	**0.0001**	**0.0001**	**0.0019**
fed TWD/fmt TWD	Pre-DSS vs. Recovery	**0.0118**	**0.0146**	**0.0001**
fed TWD/fmt TWD	Pre-DSS vs. Terminal	0.5775	0.6378	**0.0081**
fed TWD/fmt TWD	Colitis vs. Recovery	0.1399	0.1632	0.6640
fed TWD/fmt TWD	Colitis vs. Terminal	**0.0060**	**0.0067**	0.9968
fed TWD/fmt TWD	Recovery vs. Terminal	0.4997	0.4828	0.8651

Values shown are the Tukey HSD post-hoc comparison *p*-values for each pairwise comparison across time points within each experimental diet group following a generalized linear model analysis.
